# Costs of task allocation with local feedback: Effects of colony size and extra workers in social insects and other multi-agent systems

**DOI:** 10.1371/journal.pcbi.1005904

**Published:** 2017-12-14

**Authors:** Tsvetomira Radeva, Anna Dornhaus, Nancy Lynch, Radhika Nagpal, Hsin-Hao Su

**Affiliations:** 1 Electrical Engineering and Computer Science Department, Massachusetts Institute of Technology, Cambridge, MA, USA; 2 Department of Ecology and Evolutionary Biology, The University of Arizona, Tucson, AZ, USA; 3 School of Engineering and Applied Sciences, Harvard University, Cambridge, MA, USA; University of California Irvine, UNITED STATES

## Abstract

Adaptive collective systems are common in biology and beyond. Typically, such systems require a task allocation algorithm: a mechanism or rule-set by which individuals select particular roles. Here we study the performance of such task allocation mechanisms measured in terms of the time for individuals to allocate to tasks. We ask: (1) Is task allocation fundamentally difficult, and thus costly? (2) Does the performance of task allocation mechanisms depend on the number of individuals? And (3) what other parameters may affect their efficiency? We use techniques from distributed computing theory to develop a model of a social insect colony, where workers have to be allocated to a set of tasks; however, our model is generalizable to other systems. We show, first, that the ability of workers to quickly assess demand for work in tasks they are not currently engaged in crucially affects whether task allocation is quickly achieved or not. This indicates that in social insect tasks such as thermoregulation, where temperature may provide a global and near instantaneous stimulus to measure the need for cooling, for example, it should be easy to match the number of workers to the need for work. In other tasks, such as nest repair, it may be impossible for workers not directly at the work site to know that this task needs more workers. We argue that this affects whether task allocation mechanisms are under strong selection. Second, we show that colony size does not affect task allocation performance under our assumptions. This implies that when effects of colony size are found, they are not inherent in the process of task allocation itself, but due to processes not modeled here, such as higher variation in task demand for smaller colonies, benefits of specialized workers, or constant overhead costs. Third, we show that the ratio of the number of available workers to the workload crucially affects performance. Thus, workers in excess of those needed to complete all tasks improve task allocation performance. This provides a potential explanation for the phenomenon that social insect colonies commonly contain inactive workers: these may be a ‘surplus’ set of workers that improves colony function by speeding up optimal allocation of workers to tasks. Overall our study shows how limitations at the individual level can affect group level outcomes, and suggests new hypotheses that can be explored empirically.

## Author summary

Many complex systems have to allocate their units to different functions: cells in an embryo develop into different tissues, servers in a computer cluster perform different calculations, and insect workers choose particular tasks, such as brood care or foraging. Here we demonstrate that this process does not automatically become easier or harder with system size. If more workers are present than needed to complete the work available, some workers will always be idle; despite this, having surplus workers makes redistributing them across the tasks that need work much faster. Thus, unexpectedly, such surplus, idle workers may potentially significantly improve system performance. Our work suggests that interdisciplinary studies between biology and distributed computing can yield novel insights for both fields.

## Introduction

Many systems in biology and engineering, from cells to mobile networks and human societies, consist of several or many interacting units that contribute ‘work’ towards a central goal [1–6]. Each of these systems employs a ‘task allocation mechanism’, i.e., individual workers choose, or are allocated to, a specific part of the total workload, a task, which they then attempt to complete. The simplest such task allocation mechanism might be one where each individual picks a task randomly; another simple (from an algorithm standpoint) mechanism might be one where each individual is preprogrammed to always pick a defined task. For example, in a simple multicellular organism such as the alga Gonium [7], each cell processes nutrients that it happens to encounter, and each cell is equally likely to reproduce. Conversely, a car may be made up of lots of elements that need to work together to make the car run, but these elements have no flexibility with regard to how they contribute to this goal: each part fulfills its preprogrammed and unchangeable function. However, most biological systems, and many engineered ones, do not behave according to either of these extremes. Instead, individuals have to choose how to contribute, and may use various types of information about the need for different types of work to make this choice (note that we are using the term ‘choice’ in the sense of possessing an algorithm that leads to task selection, and do not imply free will). The goals of any such task allocation mechanism are to achieve efficiency and robustness of system function. For example, in a developing embryo, multiple cells have to select which organs or tissues to develop into [8]. The task allocation mechanism used has to ensure that the right cells are allocated to all necessary organs; at the same time, it has to tolerate the occasional loss of cells. Similarly, in cloud computing, the demand for different types of computation may change dynamically over time, and so might the availability of individual processors [9, 10]. The ideal task-allocation mechanism used here again has to achieve a match of allocated processors with current needs, which likely requires repeated re-allocation.

Is task allocation a difficult problem, and does it matter which algorithm is chosen? If task allocation is an easy problem, then the match of work to workers should be close enough to the theoretical optimum that the efficiency and robustness of the evolved biological systems and designed/engineered systems are not substantially reduced. However, there is evidence from theoretical computer science that indicates that task allocation (referred to as ‘resource allocation’) is difficult [11–13] in that it requires a non-negligible amount of resources (such as time, memory, and/or communication messages). In particular, [12] shows that if individuals also differ in how well they can perform different types of work, then in the model they consider, task allocation is an NP-hard problem. Another line of evidence for the idea that task allocation is difficult is the number of workers in distributed systems that are in fact not allocated to any tasks [14]. In social insect colonies in particular, a large fraction of workers do not appear to work [15]; in addition, at any point in time, there is another substantial group of individuals who are thought to be actively looking for work [16]. This may indicate either that these workers are in excess of the number needed to perform tasks, or that they are result of a task allocation mechanism that either costs time (in the form of workers looking for work) or produces inadequate allocation (unemployed workers that could be employed). Either way, this would indicate that task allocation is not an easy problem (several other hypotheses, unrelated to task allocation, have also been proposed [15]). In distributed computing, extra computing devices (in addition to the number necessary to complete the tasks) are often used to achieve fault tolerance and increase efficiency by replicating information and computation over multiple devices [9, 10]. Both of these phenomena might indicate that task allocation is neither effective nor fast: if task allocation were easy to achieve quickly, then there would not be a need for costly buffering. If task allocation is a difficult problem, we would expect to see complex systems employ imperfect mechanisms that lead to approximate solutions, or which sometimes fail to allocate workers to tasks correctly, or we might see additional strategies that compensate for mistakes of imperfect task allocation, or trade-offs between the resources invested and the quality of task allocation achieved. Thus, in these cases we expect the chosen task allocation mechanism to contribute significantly to system performance or biological fitness. It will not then be possible to understand the evolution of system organization, or to design an efficient and robust system, without also understanding the constraints imposed by the process of task allocation.

Here we aim to contribute to an understanding of what limits flexible and robust task allocation. To do this, we develop a model of task allocation in social insect colonies. We are specifically interested first in how group size, i.e. the number of individuals that may be allocated to work, affects the difficulty of correct task allocation, and second, in the effects of having more workers available than work (which would lead to inactive workers). We also discuss the effect of the number of distinct task types to which workers have to be allocated. We quantify performance of three generalized task allocation mechanisms that differ in the amount of information available to workers about the demand for work in different tasks. We are thinking of our model as representing individual insect workers making choices among such tasks as foraging or brood care. However, our model is kept general in many respects, and is thus likely to apply to many similar systems where individuals are making choices about tasks using local information.

Group size is typically thought to be a central factor in determining complex system function [17]: multicellular organisms [18], human societies and organizations [19, 20], and social and computer networks [21] all have been argued to develop more complexity, acquire new functionalities, and be competitively superior at larger group sizes, and all of this has also been argued for social insect colonies [22]. In many cases, although not unequivocally [19, 22], larger group size has been associated with more specialized, and possibly less flexible, individuals within the group; this may result from the smaller variance typically experienced by larger groups because of the ‘law of large numbers’ [23]. Larger groups may also benefit from ‘economies of scale’ when there are fixed costs that do not scale linearly with the number of individuals [24]; for example, broadcast signals reach more individuals in larger groups at the same cost [25]. Biological accounts of the evolution of larger groups, at any level of organization, typically focus on these benefits of group size [17]. In computer science, on the other hand, research has often focused on the costs of group size [13, 26]. Generally speaking, algorithms that require interactions between individuals take much longer to execute in larger groups, because the number of possible interactions increases faster than linearly with group size (with *N*^2^ for pairwise interactions, exponentially when any number of interactants is possible). Indeed this effect of group size on ‘naive’ distributed problem-solving algorithms is so great that the group size is typically equated with ‘problem size’, and the performance of algorithms is measured mainly in terms of how strongly they depend on group size or other measures closely related to group size [13, 27]. This makes sense if one assumes that the effect of group size will outweigh the effects of any constant factors on the performance of the algorithm, even for moderately large groups.

As stated above, we are using social insect colonies as a model system to study the effect of group size on the difficulty of task allocation. Social insects such as bees, ants, wasps, and termites typically live in colonies that contain one or a few queens, who are the source of colony reproduction, and many, anywhere from a handful to millions of workers, who do not reproduce but complete all other tasks [28–30]. These tasks include foraging (finding and collecting food), nest building and repair, brood care (caring for immature individuals; Hymenopteran insects such as bees and ants spend ≈ 10 − 30% of their lifespan in an immature stage in which they cannot move and have to be cleaned, fed, defended, and kept at a tolerable temperature much like the most dependent mammals in their infant stage), colony defense, and various other tasks that may include thermoregulation (such as by ventilation or heating), nest cleaning, undertaking (removing dead individuals), etc. [15]. The need for work in these different tasks typically fluctuates in daily and seasonal patterns as well as stochastically [31].

Social insect colonies are self-organized, meaning that neither the queens nor any other workers ‘direct’ the task choices of other workers, although interactions between individuals such as communication signals and aggression may affect task selection [29, 32]. There are more than 10000 species of ants alone, and different species of social insects may use different task allocation mechanisms. Any task allocation mechanism consists of two parts: the traits of individuals that predispose them to particular tasks, and the behavioral rules that lead them to select a particular task at a given moment (the individual-level algorithm; [33]). In social insects, body size, age, physiological and nutritional status, sensory abilities, and other internal factors are thought to create variation among individuals in task preferences and skills; in addition, individual experience, interactions with other workers, spatial and hierarchical position in the colony, and random encounters with tasks will do so as well, in the short and long term [14, 32, 34]. In different species, some or all of these factors may play a role in task allocation, and to differing degrees. The behavioral rule set, i.e. the algorithm, by which individuals choose a task to work on in the moment, is typically thought to involve a comparison between an individual’s task preferences and the need for a particular task; this is sometimes referred to as the ‘task stimulus response threshold mechanism’ (because workers are thought to have different thresholds at which they decide to work on a task, depending on the level of ‘task stimulus’ which communicates demand for work in the task, [35]). However, it is worth noting that the actual precise algorithm is seldom defined in the insect literature; e.g. ‘thresholds’ may actually be continuous probabilistic functions, and it is unclear how multiple task stimuli are evaluated (in random order, or at the same time, and do they interact or not). It is also typically unclear how the factors listed above interact to produce variation in preferences across tasks or across individuals; e.g. are the preferences for different tasks independent of one another or not [36]. All of this may also vary across species.

Despite this uncertainty about the precise mechanism, the fact that social insects achieve task allocation is well studied. Workers in a colony specialize to a large or small degree on different tasks, and may switch tasks as needed [37], although this may come at additional cost [38]. Colonies are typically able to effectively compensate for worker loss ([36], although see [39]) or changes in demand for different tasks [14]. However, it is also the case that inactive workers are common: at any point, often > 50%, sometimes > 70%, of the colony appear not to be performing any tasks [15]. This may be in part due to need for rest, selfish reproduction by workers [40, 41], or immaturity of workers [42]; but it has also been suggested that completely inactive and ‘walking’ (without apparently getting anything done) workers may either be looking for work and failing to find it [16], or in fact be a surplus of workers not necessary to complete the work of the colony [14]. Inactive workers, i.e. units within a complex system that are not contributing, may also be common elsewhere, both in biology and engineering [43, 44]. Here we examine the effect of such a buffer of apparently redundant workers on task allocation efficiency.

This study aims to contribute to understanding why social insects evolved the task allocation mechanisms that they did, and, more generally, what limits effective task allocation in distributed sytems. We contribute to these aims by measuring the performance of task allocation mechanisms under different assumptions. To achieve this, we derive how quickly task allocation can be achieved using distributed computing theory methods to analyze algorithm performances. We use a generalized task allocation mechanism with three different assumptions about how individual workers can acquire information about the need for more work in specific tasks (what we call the ‘deficit’). This approach then leads us to insights about whether and how task allocation is limited by group size, the relationship of group size to the total need for work (what we call the ‘demand’), the information available to workers, the number of tasks, and how precisely the colony must match the allocation of workers to demands for work across tasks. The rest of this paper is organized as follows: in the Methods section, we describe the tools and techniques we use from distributed computing theory, together with a formal model of the task allocation system we consider; in the Results section, we mathematically derive bounds (that is upper limits) on the time for ants to allocate themselves to tasks in the various versions of our formal model, and also provide some intuitive explanations and numerical examples of the results; in the Discussion section, we emphasize the implications of our results for actual ant and bee species and we address some caveats and open questions.

## Methods

In this paper, we use modeling and analysis techniques from the field of theoretical distributed computing to study the difficulty of task allocation in insect colonies. Distributed computing is a field that typically studies networked computers that jointly, but in a self-organized manner, solve a computational problem [13]. Similar to biological complex systems, the individual computers may pass messages to each other, but will be otherwise acting independently. We believe that many of the insights and tools from the field of distributed computing theory will be directly useful and informative for biology, and some recent studies have started to apply them to biological problems ([12, 45–49]).

In distributed computing theory and in this paper, models are generally abstract, discrete and probabilistic; moreover, they are modular in that each individual is modeled independently from other individuals, from the environment (including the tasks), and from the information about tasks the environment may provide to individuals. In these models, we design distributed algorithms and assign an independent copy of the algorithm to run at each individual. We analyze the algorithms mathematically, using proof techniques from probability theory and algorithm complexity, to derive guarantees on the solvability and efficiency of task allocation (measured as the time for workers to allocate themselves correctly to tasks). The specific results we present have both a worst-case and an average-case flavor. The worst-case aspect of the results refers to the possible initial values of the parameters in the system; in other words, we do not measure the performance of our algorithms with respect to the expected average performance given some distribution of starting environments, but instead consider how well the algorithm will do with the worst possible starting conditions (e.g. with respect to the distribution of demands across different task types). The average-case aspect of the results is with respect to the probability distribution of the actual decisions of the workers and the probabilistic feedback they receive from the environment. We elaborate more on this distinction in the Informal Definitions and descriptions section.

### Our approach

The specific abstraction of the task allocation problem that we study involves a distributed process of allocating all workers to tasks with the goal of satisfying the demand for each task. The demand for each task can be thought of as a work-rate required to keep the task satisfied. We consider all workers to be equal in skill level and preferences. While this is an abstraction, we focus here on simply the challenge of allocating generalist workers among tasks. We do not attempt to model how the demand for a task is computed or measured empirically. Instead, we assume that as a result of workers trying to maximize the fitness of the colony, there is some optimal number of workers performing each task, and this is what the workers should attempt to match.

At each time step, each worker decides what task to work on based on simple feedback from the environment informing the worker of the state of the tasks. In particular, we consider two specific types of environment feedback: (1) whether the worker is successful at its current task, and (2) which task does the work choose next. We analyze whether this general algorithm is able to successfully allocate the workers so that all tasks are satisfied, and the time for this process to terminate. In particular, we focus on upper bounds for the time to satisfy all tasks (i.e. how long it is expected to take given the worst possible starting conditions) as a function of colony size, the number of tasks, and the total amount of work in the presence or absence of extra workers (beyond the minimum to satisfy all tasks) in the colony.

### Informal definitions and descriptions

#### Model

We consider a setting in which all workers are identical and each worker can supply one unit of work to each task type (brood care, foraging, nest maintenance, etc.). For brevity, for the rest of the paper, we will refer to tasks types as *tasks*.

At the start of the re-allocation process, each task is characterized by an integer-valued demand, and we consider a task to be satisfied when the number of units of work provided to the task is at least as much as the demand of the task. In order to guarantee that it is possible to satisfy the demands of all tasks, we assume that the number of workers is at least as large as the total sum of all demands.

We also assume the workers perform actions in lock step and that each such step is sufficiently long so that the workers can re-evaluate the state of the environment at the end of each round, which includes the effect of the work performed by other workers in that round. Based on that information, at the end of each step, each worker decides what action to perform (what task to work on) in the next step. We measure the efficiency of the re-allocation process as the number of steps necessary for the workers to re-allocate to the tasks in a way that matches or exceeds the demands (we term this ‘successful reallocation’).

#### Feedback about task demands

We abstract away from actual low-level mechanisms that workers use to acquire knowledge about the environment; instead, we focus on the *information content* of the environment feedback. Therefore, we can model feedback that is minimal and probabilistic. Our goal is to provide only limited information about the state of the environment.

In particular, we consider environment feedback that consists of two components: *success* and *choice*. The first component, *success*, informs each worker whether it is *successful* at the task it is currently working on (i.e. whether its work was needed there), and the second component, *choice*, provides each worker with an alternative task to work on, in case it is not successful at its current task. From a biological perspective, the separation between these two components is motivated by the two main ways a worker interacts with its environment: (1) from attempting to work on some task, a worker learns whether its work is needed, and (2) from randomly interacting with tasks in the nest, it may perceive need for work in tasks it is not active in. We consider the following specifications for *success* and *choice*.

*Success feedback:* We assume that for a given time step and a given task, if the number of workers working on this task is less than or equal to the demand of the task, then all workers working on the task are ‘successful’. Otherwise, if more workers are working on the task than the demand requires, then we assume *success* informs only as many workers as needed to satisfy the demand that they are successful, and it informs the rest of the workers working on the task that they are unsuccessful. Since workers are identical and do not store any work history (similarly to a Markov process), it is not important which workers are the successful ones and which workers are the unsuccessful ones among all the workers working on some task, as long as the number of successful workers does not exceed the demand of the task.

A good analogy to the *success* feedback is the game of musical chairs: the number of chairs corresponds to the demand of the task, and the number of workers working on the task corresponds to the number of people playing the game. In musical chairs, all players who manage to find a seat when the music stops continue to the next round; similarly, the workers that manage to complete some amount of work that contributed to decreasing the demand are considered successful.

As a result, *success* provides each worker with implicit information about the amount of work needed for the task without directly informing the worker of the exact value of that amount.

*Choice feedback:* For the second component, *choice*, of the environment feedback, we assume workers determine an alternative task to work on by encountering tasks randomly. We model three probability distributions for the *choice* component.

The simplest way to model a worker encountering a random task in the nest is to assume *choice* provides the worker with (1) a uniformly random task (that is, each task is equally likely to be chosen). We think of the uniform distribution as a very natural way to choose a task without any information about the set of tasks or their demands. Other distributions imply some knowledge about parameters of the distribution. For example, the normal distribution implies we have some information about the mean and variance of the distribution. Even more importantly, since our random variable is discrete, the normal distribution is not a good choice because we do not assume any ‘ordering’ of the tasks. Thus, the ‘uniform’ distribution here simply means that each task is chosen with equal probability. Alternatively, we might think workers recognize tasks that need work, and *choice* might provide (2) a uniformly random task only among the unsatisfied tasks. Finally, we might think that tasks provide information on their level of demand, and thus workers may be able to choose (3) a task that needs more work compared to other tasks. Option (1) implies that workers initially choose a task with no information on the demand for work in different tasks. Options (2) and (3) imply that workers can sense which tasks need work before engaging in them, e.g. through a task stimulus produced by unsatisfied tasks such as pheromone produced by hungry (unfed) brood (indicating need for brood care). Since we assume that in all cases workers will discover whether their contribution was actually needed through the ‘success feedback’ mechanism, options (1) and (2) imply that tasks are ultimately checked for demand one at a time, i.e. with a cost of one round per task checked, while in option (3) workers can sense demand for all tasks at once.

See Table 1 for an example execution of the task allocation system.

**Table 1:** Sample execution of a task allocation in our model.

	Inactive Workers	Task 1 ⋆⋆	Task 2 ⋆ ⋆ ⋆⋆	Task 3 ⋆	Task 4 ⋆ ⋆ ⋆
Time 0:	• • • • • • • • • • ••	∘∘	• ∘ ∘∘	∘	• ∘ ∘
Time 1:	• • • • • • • • ••	•∘	• ∘ ∘∘	•	• ∘ ∘
Time 2:	• • • • ••	•∘	• • ∘∘	•	• • ∘
Time 3:	• • ••	•∘	• • ∘∘	• • •	• • ∘
Time 4:	••	••	• • ••	•	• • •

Sample execution of a task allocation in our model. The stars denote the demand of each task, the empty circles denote unsatisfied units of work, and the solid circles denote workers working on specific tasks. The execution begins at time 0 when only two workers are working on tasks 2 and 4. Then, at time 1 some workers join tasks 1 and 3. At time 2, more workers join all tasks. At time 3, too many workers join Task 3 and only one of them is successful because the demand for the task is 1. Finally, at time 4 all tasks are satisfied. The remaining workers indicate that the size of the colony is greater that the total sum of the demands of all tasks.

#### Performance measure

In all three of the options for the *choice* component, keeping the *success* component the same, we are interested in upper bounds on (that is, the maximum value of, and thus the worst-case for) the time until workers are correctly re-allocated such that the demands of the tasks are satisfied. It is important to note that our results have both a worst-case flavor (in terms of the initial configuration of the system) and average-case flavor (in terms of the probability distribution defined by the *choice* component).

The worst-case analysis refers to the initial assignment of workers to tasks as well as the demands of the tasks. So, when we say that for some scenario the running time is at most *t*, informally, it implies that for any possible initial configuration of task demands and assignment of workers to tasks, starting from that configuration, it takes time at most *t* to re-allocate the workers correctly. It is not always clear whether there exists an initial configuration (assignment of workers to tasks and task demands) that results in a re-allocation of exactly time *t*; it is also not straightforward to identify the initial configuration that requires the most rounds for workers to re-allocate correctly (the ‘worst-case’ initial configuration). In other words, we do not average the time to re-allocate over all possible initial configurations. Averaging over all possible initial configurations would be a challenging task given that the space of such initial configurations is very large; moreover, we would have to assume all initial configurations are equally likely to arise, which may not necessarily be a reasonable assumption.

The average-case (or more generally, probabilistic) analysis refers to the fact that we use the distribution of outputs of the *choice* component. So, when we say that for some scenario with probability at least *p* the running time is at most *t*, informally, it implies that we took into account all possible outputs of *choice* and their likelihood in order to calculate *t*. In other words, it is possible that the workers do not re-allocate within time *t* (or ever), but the probability of that happening is less that 1 − *p* (usually extremely small). Analyzing the running time in such a probabilistic way is a manageable task because we know exactly what the distribution of outputs of *choice* is for each of the three options and at each step.

### Formal definitions

See S1 Text for a more detailed version of this section.

Let *A* denote the set of workers and *T* denote the set of tasks. Each task *i* ∈ *T* has an integer demand *d*_*i*_ that represents the minimum number of workers required to work on task *i* in order to satisfy the task. Let *w*_*i*_ denote the total number of worker units of work currently supplied to task *i*. Let $\vec{w}$ and $\vec{d}$ denote the vectors of *w*_*i*_ and *d*_*i*_ values, respectively, for each 1 ≤ *i* ≤ |*T*|. The $\vec{d}$ vector is static, while $\vec{w}$ changes over time depending on the different tasks workers choose to work on. Clearly, in order for all demands to be met, there should be sufficiently many workers in the colony. We assume that there exists a real *c* ≥ 1 such that |*A*| = *c* ⋅ ∑_*i*∈*T*_ *d*_*i*_.

#### Feedback

We consider two feedback components, *success* and *choice*, that provide each worker with a boolean in {0, 1} and a task in *T* ∪ {⊥}, respectively, determined based on $\vec{w}$ and $\vec{d}$. The output values of *success* and *choice* are determined according to some probability distributions.

#### Workers

Each worker *a* ∈ *A* has a state *q* ∈ *Q* = {*q*_⊥_, *q*_1_, *q*_2_, ⋯, *q*_|*T*|_} at each point in time, where *q*_⊥_ indicates that worker *a* is not working on any task and each state *q*_*i*_, for *i* ∈ {1, ⋯, |*T*|}, indicates that worker *a* is working on task *i*. Each worker is modeled as a finite state machine with transition function *δ*: *Q* × ({0, 1} × (*T* ∪ {⊥})) → *Q*; in other words, each worker’s new state is determined by its old state and its inputs from the *success* and *choice* components. Let *q* be the current state of some worker *a*, and let *q*′ be the resulting state of worker *a* after applying *δ*. In each step, *q*′ is determined as follows: *q*′ = *q* if *success* outputs 1, and *q*′ = *q*_*i*_ if *success* outputs 0 and *choice* outputs *i* ∈ *T* ∪ {⊥}.

#### Execution

The execution of any algorithm solving the task allocation problem starts at time 0 and proceeds in synchronous rounds, such that each round *r* + 1, for *r* ≥ 0, denotes the transition from time *r* to time *r* + 1. In each round *r* + 1, the *success* and *choice* components provide each worker with a boolean and a task. Each worker component performs a state transition using its *δ* transition function and performs some amount of work on the task associated with its state.

#### Problem statement

A task *i* ∈ *T* is *satisfied* at time *r* if *d*_*i*_ ≤ *w*_*i*_(*r*). An algorithm satisfies all tasks by time *r* ≥ 0 if for each *r*′ ≥ *r*, all tasks *i* ∈ *T* are satisfied at time *r*′.

The specification of *success* and some of the specifications of *choice* in this section are inspired by the biological model by Pacala et al. [50] and simplified for the sake of easier analysis.

#### Success component

The *success* component determines whether each worker is successful at the task it is currently working on and allows excess workers working on a satisfied task to switch to another task. Throughout this paper, we consider *success* components that satisfy the following conditions in each execution and at each time *r* of the execution: for each task *i* ∈ *T*, |{*a*∣*a* is in state *q*_*i*_ at time *r* and receives 1 from the *success* component in round *r* + 1}| = min(*d*_*i*_, *w*_*i*_(*r*)). Also, each worker in state *q*_⊥_ at time *r* receives 0 from *success* in round *r* + 1.

#### Choice component

The *choice* component returns a candidate task to each worker as an alternative task to work on. We consider three different specifications of *choice*:

1. *choice* returns a task drawn from all the tasks in *T* uniformly at random (with probability 1/|*T*|).
2. *choice* returns a task drawn from the set of unsatisfied tasks, *U*(*r*) = {*i*∣*d*_*i*_ > *w*_*i*_(*r*)}, uniformly at random. If there is no such task, then *choice* returns ⊥.
3. *choice* returns a task *i* drawn from the set of all unsatisfied tasks with probability (*d*_*i*_ − *w*_*i*_(*r*))/∑_*j*∈*U*(*r*)_(*d*_*j*_ − *w*_*j*_(*r*)). This option corresponds to the scenario where workers can somehow sense the need to work on each task, and are more likely to work on tasks with high deficit *d*_*i*_ − *w*_*i*_(*r*) compared to the total deficit of all unsatisfied tasks ∑_*j*∈*U*(*r*)_(*d*_*j*_ − *w*_*j*_(*r*)).

## Results

First, we present the formal statement of our results, together with simple proof overviews. We start by introducing a few general facts about the task allocation system, like properties of the *success* and *choice* feedback, and simple results about the the general growth of the level of satisfaction of each task. Next, we describe the main results corresponding to each of the three options for the *choice* components. For each such option, we present the formal result on how much time is required for workers to correctly re-allocate, and then describe informally the main arguments of the proofs. The full proofs of all the results are available in S2 Text. Readers uninterested in the specific mathematical arguments can skip to the Non-technical Summary of Results section. Finally, in the Numerical results section, we provide numerical examples that illustrate our results with respect to concrete values of the parameters.

### General facts

In this section, we give some basic definitions and results that will be used in the subsequent analyses of the convergence times for the various *choice* options.

A task is *satisfied* at time *r* if *d*_*i*_ ≤ *w*_*i*_(*r*). Let *S*(*r*) denote the set of satisfied tasks at time *r*. Let *U*(*r*) = *T* \ *S*(*r*) denote the set of unsatisfied tasks at time *r*. For each task *i* ∈ *T* and each time *r*, let Φ_*i*_(*r*) = max{0, (*d*_*i*_ − *w*_*i*_(*r*))} be the *deficit* of task *i* at time *r*. If *i* ∈ *U*(*r*), then Φ_*i*_(*r*) = *d*_*i*_ − *w*_*i*_(*r*). We define the *total deficit* at time *r*:

$$\begin{array}{c} \Phi \left(r\right)=\sum_{i\in T}\Phi_{i}\left(r\right). \end{array}$$

Define a worker to be *inactive* in round *r*, for *r* > 0, if it is in state *q*_⊥_ at time *r* − 1 or if it receives 0 from *success* in round *r*. In other words, a worker is inactive if it is not working on any task, or if it unsuccessful at the current task it is working on.

For a full list of the parameters used in the model and analysis, see Table 2.

**Table 2:** Summary of parameters in the task allocation model and analysis.

Symbol	Parameter definition	Plausible range	Explanation for range	References
|*T*|	number of tasks	[2, 20]	At low end if conceived of as the number of distinct worker task groups; at higher end if all ‘identifiable’ worker activities are included.	[15, 51–53]
Φ	initial deficit	[5, 500]	Considerable variation across species and situations; what is empirically measured is the number of workers actually re-allocated or activated.	[31, 54–57]
|*A*|	number of workers	[2, 20 million]	Most species are in the 10-500 range for total colony size.	[22]
*D*	total task demands	[2, 20 million]	We assumed here that the demand for work, measured in insect workloads, is in the same range as the colony size (see section 4.3 for discussion).	[22]
*c*	extra workers (|*A*|/*D*)	[1, 2]	Since *D* has not been empirically measured, neither has *c*. If we assume ‘inactive’ workers may be in excess of work that needs to be performed, values in the entire range are plausible.	[15, 52, 58–60]
1 − *δ*	success probability	[0.5, 0.95]	To our knowledge, no attempts to estimate delta or epsilon exist. Our estimates are simply based on the assumption that in some cases, e.g. defense, colonies would need to be ‘very’ certain that approximately the correct number of workers are allocated to the task at hand; in other cases, such as foraging, colonies may only need moderate certainty that task allocation is successful.	
1 − *ϵ*	fraction of deficit to be satisfied	[0.7, 0.9]	*ϵ* reflects the degree to which the demand for work in a task is exactly matched. Given the high degree of stochasticity observed in task allocation in social insects, we assumed here that 1 − *ϵ* is not required to be ‘very’ close to 1 in most cases.	[54, 61]

Based on the basic properties of the *success* and *choice* components, we can establish the following facts:

1. The number of work units supplied to a given task *i* ∈ *T* is non-decreasing.
2. For each *r* ≥ 0, |*U*(*r*)| ≥ |*U*(*r* + 1)| and |*S*(*r*)| ≤ |*S*(*r* + 1)| (follows from fact 1). In other words, the number of unsatisfied tasks never increases and the number of satisfied tasks never decreases.
3. For each *r* ≥ 0, Φ_*i*_(*r*) ≥ Φ_*i*_(*r* + 1). The deficit of each task never increases.
4. By the assumption that |*A*| = *c* ⋅ ∑_*i*∈*T*_ *d*_*i*_, the number of inactive workers in round *r* + 1 is at least *c* ⋅ Φ(*r*). So, the more total deficit, the more inactive workers we have.
5. If the probability to satisfy a task in round *r* + 1 is at least *p*, then E[|U(r+1)|]≤|U(r)|⋅(1−p) and E[Φ(r+1)]≤Φ(r)⋅(1−p). In other words, if we know the probability with which each task gets satisfied in a given round, we can calculate the expected number of unsatisfied tasks and the expected total deficit in the next round.
6. If *choice* always returns an unsatisfied task to each worker, then the workers re-allocate successfully in at most |*T*| rounds.

Next, we analyze the three variations of the *choice* component.

### Uniformly random tasks

In this section, we consider the first option for the *choice* component, where in each round *choice* returns a task *i* with probability 1/|*T*|. This section includes only proof overviews and approximate running times. For detailed proofs of the results in this section, refer to S2 Text.

One of the main results for this option of the *choice* component states that for any success probability 1 − *δ* that we choose, the time until workers re-allocate correctly is at most $O(|T|c^{-1})(\text{ln}\;\Phi (0)+\text{ln}(1/\delta ))$. We can see the time is linearly proportional to the number of tasks |*T*|, logarithmically proportional to the total amount of work needed (Φ(0)) and the inverse of the failure probability, and inversely proportional to *c*, the ratio of the colony size to the total sum of demands of tasks.

**Theorem 1.** *For any δ*, 0 < *δ* < 1, *with probability at least* 1 − *δ, all tasks are satisfied by time* $O(|T|c^{-1})(\text{ln}\;\Phi (0)+\text{ln}(1/\delta ))$.

*Proof Idea:* We know that the number of inactive workers in round *r* + 1 is at least *c* ⋅ Φ(*r*) (by fact 4). By the definition of *choice* in this section, each inactive worker starts working on each task *i* with probability 1/|*T*|. Therefore, we can show that, in each round, the expected number of new workers to join each unsatisfied task is at least *c* ⋅ Φ(*r*)/|*T*|.

First, consider the case when *c* ≤ 2|*T*| and consider some time *r*. After some workers join task *i* in round *r* + 1, it is not guaranteed that the entire new set of workers remains working on task *i* because some workers may be unsuccessful if task *i* does not require that many workers. Assuming *c* ≤ 2|*T*|, since the total deficit is Φ(*r*) and there are |*T*| tasks, we can show that in expectation the total deficit in the next round is at least *c* ⋅ Φ(*r*)/|*T*| (which can be 0 if all tasks are satisfied). Therefore, in expectation, at least *c* ⋅ Φ(*r*)/|*T*| of the new workers that join tasks will remain working on them. This implies that the expected total deficit Φ(*r*) decreases by approximately *c* ⋅ Φ(*r*)/|*T*| in round *r* + 1.

Next, we consider the case of *c* > 2|*T*|. We can express *c* as a multiple of |*T*|: *c* = *c*′ ⋅ |*T*| for some *c*′ > 2. We can show that in each round, the probability to satisfy each task is at least some constant, and consequently (using fact 5 above), we conclude that the expected number of unsatisfied tasks and the total deficit decrease by a constant fraction in each round.

Finally, we start at time 0, when the total deficit is Φ(0), and inductively apply the conclusions above in the cases of *c* ≤ 2|*T*| and *c* > 2|*T*|. By facts 2 and 3, we know that both |*U*| and Φ are non-increasing, so we just need to analyze how fast they decrease. For the case of *c* ≤ 2|*T*|, the expected total deficit Φ(*r*) decreases by approximately *c* ⋅ Φ(*r*)/|*T*| in each round *r* + 1. So it will take approximately (|*T*|/*c*) ln Φ(0) rounds until the total deficit decreases to 0. To turn this into a more formal probabilistic claim, we can add approximately ln(1/*δ*) rounds, for some 0 < *δ* < 1, in order to ensure that the tasks are satisfied not only in expectation, but with probability at least 1 − *δ*. This trick works by applying a simple Markov bound (see S2 Text).

The second main result for this option of the *choice* component studies the time until workers re-allocate in such a way that, for any success probability 1 − *δ* and any fraction *ϵ* that we choose, a (1 − *ϵ*)-fraction of the total work Φ(0) is satisfied with probability at least 1 − *δ*. The time to re-allocate in this case is at most $O(|T|c^{-1})(\text{ln}(1/\epsilon )+\text{ln}(1/\delta ))$. Similarly to the first result in this section, the time is linearly proportional to the number of tasks |*T*|, logarithmcally proportional to the inverse of the failure probability, and inversely proportional to *c*, the ratio of the colony size to the total sum of demands of tasks. However, here, we do not have a dependence on Φ(0), but only a logarithmic dependence on 1/*ϵ*.

**Theorem 2.** *For any δ and ϵ*, 0 < *δ, ϵ* < 1, *with probability at least* 1 − *δ, the deficit at time* $O(|T|c^{-1})(\text{ln}(1/\epsilon )+\text{ln}(1/\delta ))$ *is at most ϵ* ⋅ Φ(0).

*Proof Idea:* Following the same structure as the proof above, we can also compute the number of rounds until the tasks are satisfied approximately. Suppose we only want a (1 − *ϵ*) fraction of Φ(0) to be satisfied for 0 < *ϵ* < 1. Recall that for *c* ≤ 2|*T*|, the expected total deficit Φ(*r*) decreases by approximately *c* ⋅ Φ(*r*)/|*T*| in each round *r* + 1. So it will take only (|*T*|/*c*)(ln(1/*ϵ*) + ln(1/*δ*)) rounds to ensure this is true with probability at least 1 − *δ* (again, the ln(1/*δ*) factor is to ensure the probability guarantee).

For the case of *c* > 2|*T*|, we proceed similarly. Recall that in this case *c*′ = *c*/|*T*| and the expected number of unsatisfied tasks and the total deficit decrease by a constant fraction in each round (this constant depends on *c*′). So, with probability at least 1 − *δ*, all tasks are satisfied by time approximately (1/*c*′)(min{ln |*T*|, ln Φ(0)} + ln(1/*δ*)). The reason for having a minimum is to take advantage of the smaller value between |*T*| and Φ(0). And similarly, if we only want to satisfy the tasks approximately the ln Φ(0) term turns into ln (1/*ϵ*).

### Uniformly random unsatisfied tasks

In this section, we consider the second option for the *choice* component where in each round *choice* returns a task *i* ∈ *U*(*r*) with probability 1/|*U*(*r*)|. This section includes only proof overviews and approximate running times. For detailed proofs of the results in this section, refer to S2 Text.

One of the main results for this option of the *choice* component states that for *c* ≥ 1 and any success probability 1 − *δ* that we choose, the time until workers re-allocate correctly is at most O(lnΦ(0)+ln(1/δ)). We can see the time is logarithmically proportional to the total amount of work needed (Φ(0)) and the inverse of the failure probability. Since *c* may be extremely close to 1, we do not get any effect of *c* in this result.

**Theorem 3.** *For c* ≥ 1 *and for any δ*, 0 < *δ* < 1, *with probability at least* 1 − *δ, all tasks are satisfied by time* min{|T|,O(lnΦ(0)+ln(1/δ))}.

*Proof Idea:* Suppose *c* ≥ 1 and consider some time *r*. We can show that in round *r* + 1 at least one of the following happens: (1) the total deficit decreases by a constant fraction, or (2) the number of unsatisfied tasks decreases by a constant fraction. To show the first property holds, we consider tasks with a fairly high deficit, which are not likely to get satisfied in one round. We show that the number of new workers joining such tasks is enough to decrease the total deficit by a constant fraction. To show the second property (the number of unsatisfied tasks decreases by a constant fraction), we focus on tasks with fairly low deficit which are likely to get satisfied within one round. We can show that these tasks are enough to decrease the total number of unsatisfied tasks by a constant fraction in one round. For showing both (1) and (2), we first prove a bound on the probability to satisfy any given task in a single round and then use fact 5 to get a bound on the expected number of unsatisfied tasks and the expected total deficit.

Finally, we start at time 0, when the total deficit is Φ(0) and the number of unsatisfied tasks is at most |*T*|, and inductively apply the two results above. By facts 2 and 3, we know that both |*U*| and Φ are non-increasing, so we just need to analyze how fast they decrease. If it is the case that the expected total deficit Φ(*r*) decreases by a constant factor in each round, then it will take approximately ln Φ(0) rounds until the total deficit decreases to 0. If it is the case that the number of unsatisfied tasks decrease by a constant factor in each round, then it will take approximately ln |*U*(0)| rounds until the total deficit decreases to 0. Since Φ(0) ≥ |*U*(0)|, we know either Φ(0) or |*U*(0)| will decrease to 0 in approximately 2 ln Φ(0) rounds. To turn this into a more formal probabilistic claim, we can add approximately ln (1/*δ*) rounds, for some 0 < *δ* < 1, in order to ensure that the tasks are satisfied not only in expectation, but with probability at least 1 − *δ*. This trick works by applying a simple Markov bound (see S2 Text). The minimum in the final bound follows by fact 6 in the General Facts section.

The second main result for this option of the *choice* component states that for *c* > 1 and any success probability 1 − *δ* that we choose, the time until workers re-allocate correctly is at most O(1/lnc)(ln|T|+ln(1/δ)). Similarly to the result above, the time is logarithmically proportional to the total amount of work (Φ(0)) needed initially, and the inverse of the failure probability. Now, *c* is strictly greater than 1, so we see that the time is also inversely proportional to the natural logarithm of *c*.

**Theorem 4.** *For c* > 1 *and for any δ*, 0 < *δ* < 1, *with probability at least* 1 − *δ, all tasks are satisfied by time* min{|T|,O((1/lnc)(ln|T|+ln(1/δ)))}.

*Proof Idea:* Suppose *c* > 1 and consider some time *r*. Unlike the case of *c* ≥ 1, where in round *r* + 1 either the total deficit or the number of unsatisfied tasks decreases by a constant fraction, here we can show that the number of unsatisfied tasks decreases by at least a constant fraction in round *r* + 1. We consider all tasks with a fairly low deficit, which are likely to get satisfied in a single round. The total deficit at time *r* is Φ(*r*), and the total number of inactive workers in round *r* + 1 is at least *c* ⋅ Φ(*r*). The fact that the number of inactive workers is at least a constant fraction greater than the total deficit lets us show that the expected number of low-deficit tasks is at least a constant fraction of all unsatisfied tasks. Therefore, by satisfying these low-deficit tasks the number of unsatisfied tasks decreases by a constant fraction in expectation. Again, we can show this by proving a bound on the probability to satisfy any given task and then using fact 5. The value of that constant fraction by which the number of unsatisfied tasks decreases is what determines the dependence of the running time on 1/ln *c* in this case.

Finally, we start at time 0, when the total deficit is Φ(0) and the number of unsatisfied tasks is |*U*(0)|, and inductively apply the result above to show that the workers will re-allocate correctly within O(ln|U(0)|+ln(1/δ)) rounds. Note that ln |*U*(0)| ≤ ln |*T*| and ln |*U*(0)| ≤ Φ(0). The minimum in the final bound follows by fact 6 in the General Facts section.

We can combine the results of the two theorems in this section. Clearly, if *c* is extremely close to 1, the 1/ln *c* term becomes very large, and in the limit the running time becomes ∞. Therefore, we can take the minimum of the running times in the cases of *c* ≥ 1 and *c* > 1 to get the overall running time of the algorithm. Essentially, the running time is determined mostly by the case of *c* > 1, except for the small range of values for *c* when *c* is very close to 1.

### Unsatisfied tasks prioritized by deficit

In this section, we consider the third option for the *choice* component where in each round *choice* returns a task *i* ∈ *U*(*r*) with probability (*d*_*i*_ − *w*_*i*_(*r*))/Φ(*r*). This section includes only proof overviews and approximate running times. For detailed proofs of the results in this section, refer to S2 Text.

One of the main results for this option of the *choice* component states that for *c* ≥ 1 and any success probability 1 − *δ* that we choose, the time until workers re-allocate correctly is at most O(lnΦ(0)+ln(1/δ)). We can see the time is logarithmically proportional to the total amount of work needed (Φ(0)) and the inverse of the failure probability. Since *c* may be extremely close to 1, we do not get any effect of *c* in this result.

**Theorem 5.** *For c* ≥ 1 *and for any δ*, 0 < *δ* < 1, *with probability at least* 1 − *δ, all tasks are satisfied by time* min{|T|,O(logΦ(0)+log(1/δ))}.

*Proof Idea:* Since an inactive worker starts working on a task *i* with probability (*d*_*i*_ − *w*_*i*_(*r*))/Φ(*r*), and since there are at least Φ(*r*) inactive workers in round *r* + 1, the expected number of new workers to join task *i* in round *r* + 1 is at least a constant fraction of *d*_*i*_ − *w*_*i*_(*r*), which is exactly the deficit of the task at time *r*. We can show that each task is satisfied in round *r* + 1 with probability 1/2, and so, by fact 5 the total number of unsatisfied tasks and the total deficit decreases by half in expectation. Finally, we start at time 0, when the total deficit is Φ(0) and inductively apply the observation above to show that the workers will re-allocate correctly in approximately logΦ(0) rounds. The minimum in the final bound follows by fact 6 in the General Facts section.

The second main result for this option of the *choice* component states that for *c* > 1 and any success probability 1 − *δ* that we choose, the time until workers re-allocate correctly is at most O(1/c)(lnΦ(0)+ln(1/δ)). Similarly to the result above, the time is logarithmically proportional to the total amount of work needed (Φ(0)) and the inverse of the failure probability. Now, *c* is strictly greater than 1, so we see that the time is also inversely proportional to the natural logarithm of *c*.

**Theorem 6.** *For c* > 1 *and for any δ*, 0 < *δ* < 1, *with probability at least* 1 − *δ, all tasks are satisfied by time* min{|T|,O(1/c)O(lnΦ(0)+ln(1/δ))}.

*Proof Idea:* For the case of *c* > 1, similarly to the case of *c* ≥ 1, we show that each task is satisfied with a constant probability, so the number of unsatisfied tasks and the total deficit decrease by a constant fraction in each round. The value of that constant fraction is what let us show that the running time depends on 1/*c*. The minimum in the final bound follows by fact 6 in the General Facts section.

We can combine the results the two theorems in this section. Clearly, if *c* is extremely close to 1, the 1/*c* term becomes very large, and in the limit the running time becomes ∞. Therefore, we can take the minimum of the running times in the cases of *c* ≥ 1 and *c* > 1.

#### Introducing noise

Suppose the *success* component is not completely reliable and it can flip the 0/1 bits of at most 0 ≤ *z* ≤ |*A*| workers in round *r* + 1. Moreover, we assume the information needed to determine the outputs of the *choice* component in the same round is based on the state variables at time *r*. That is, the *choice* component does not incorporate the *z* potential mistakes into its outputs. Also, suppose the *choice* component is also not completely reliable and can change the probability of outputting task *i* from exactly Φ_*i*_(*r*)/Φ(*r*) to any value larger than (1 − *y*)(Φ_*i*_(*r*)/Φ(*r*)) for any 0 ≤ *y* < 1 while still maintaining a probability distribution over all the tasks.

Although it is no longer possible to guarantee that all tasks are satisfied, we can show that the deficit does not exceed *z*, and the time to achieve this increases as *y* approaches 1. For any success probability 1 − *δ* that we choose and any noise parameters *y* and *z* (within the permitted ranges), we study the time until workers re-allocate in such a way that at most *z* units of work remain unsatisfied. Similarly to above, the time is logarithmically proportional to the total amount of work needed (Φ(0)) and the inverse of the failure probability. Additionally, for the case of *c* ≥ 1 (in particular when *c* is very close to 1) the time is inversely proportional to ln (1/*y*), a value that gets extremely large as *y* gets very close to 1. In the case of *c* > 1, the time is inversely proportional to *c* and does not have the dependence on *y*.

**Theorem 7.** *For c* ≥ 1, *for any δ*, 0 < *δ* < 1, *and for* r=min{|T|,O(1/ln(1/y))(lnΦ(0)+ln(1/δ))}, Pr[Φ(*r*) ≤ *z*] ≥ 1 − *δ*.

*Proof Idea:* Similarly to the proofs in the previous sections, we need to get a statement on how quickly the expected value of the total deficit decreases. Here, we get a similar result; however, the rate of decrease of the total deficit also depends on the parameters of the noise *y* and *z*. In particular, we can show that in each round, the expected total deficit decreases by a 1 − (3 + *y*)/4 fraction (note that this extremely small as *y* gets close to 1) and it may never go lower than *z*. The minimum in the final bound follows by fact 6 in the General Facts section.

With the above result in mind, we can apply the usual strategy of starting at time 0 when the total deficit is Φ(0) and inductively applying the claim above. The time until the workers re-allocate correctly (with the exception of at most *z* units of work) is approximately (1/ln(1/*y*))(ln Φ(0) + ln(1/*δ*)).

**Theorem 8.** *For c* > 1, *for any δ*, 0 < *δ* < 1, *and for* r=min{|T|,O(1/c)(lnΦ(0)+ln(1/δ))}, Pr[Φ(*r*) ≤ *z*] ≥ 1 − *δ*.

*Proof Idea:* Similarly to the previous sections, we can show a similar result for *c* > 1. We show that the probability to satisfy each task in each round is some constant that depends on *c* and that determines the 1/*c* factor in the running time. Then, we show that the expected total deficit decreases by a constant fraction (that also depends on *c*) and it may never go lower than *z*. Note that, unlike the case of *c* ≥ 1, here the ‘extra workers’ help cancel the effect of *y* on the running time. Finally, we start at time 0 when the total deficit is Φ(0) and inductively apply the claim above. The time until the workers re-allocate correctly (with the exception of at most *z* units of work) is approximately (1/*c*)(ln Φ(0) + ln(1/*δ*)). The minimum in the final bound follows by fact 6 in the General Facts section.

As in the previous sections, we can combine the above two theorems by taking a minimum.

### Non-technical summary of results

For the various options for the *choice* feedback component (keeping the *success* component the same), we study the time to correctly re-allocate all workers: the number of steps workers need to take until the demands of all tasks are satisfied or over-satisfied. In particular, we show three types of results, which differ in precisely what conditions are set on this performance measure (rows in Table 3).

**Table 3:** Summary of results.

	option (1)	option (2)	option (3)
satisfy all Φ work with prob. 1 − *δ*	O(|T|(1/c)) (ln Φ + ln (1/*δ*))	min{|*T*|, (min{1,O(1/lnc)}⋅ (ln Φ + ln (1/*δ*)))}	min{|T|,O(1/c) (ln Φ + ln (1/*δ*))}
satisfy Φ(1 − *ϵ*) work with prob. 1 − *δ*	O(|T|(1/c)) (ln (1/*ϵ*) + ln (1/*δ*))	min{|*T*|, (min{1,O(1/lnc)}⋅ (ln (1/*ϵ*) + ln (1/*δ*)))}	min{|T|,O(1/c) (ln (1/*ϵ*) + ln (1/*δ*))}
satisfy Φ − *z* work under uncertainty	did not analyze	did not analyze	min{|*T*|, (ln Φ + ln (1/*δ*)) O(max{1/c, 1/ln (1/*y*)})}

The values in the table are upper bounds on the time for workers to achieve a task allocation that fulfills the criteria in the first column, given a particular option for the choice feedback. Results are presented in ‘big O’ (asymptotic) notation, which only gives the type of dependence on particular parameters, without specifying constant factors. This helps emphasize the parameters the results depend on, and does not give any information on the exact values of the running times. For precise values of these results, see the Numerical results section and S2 Text.

**Fig 1:**
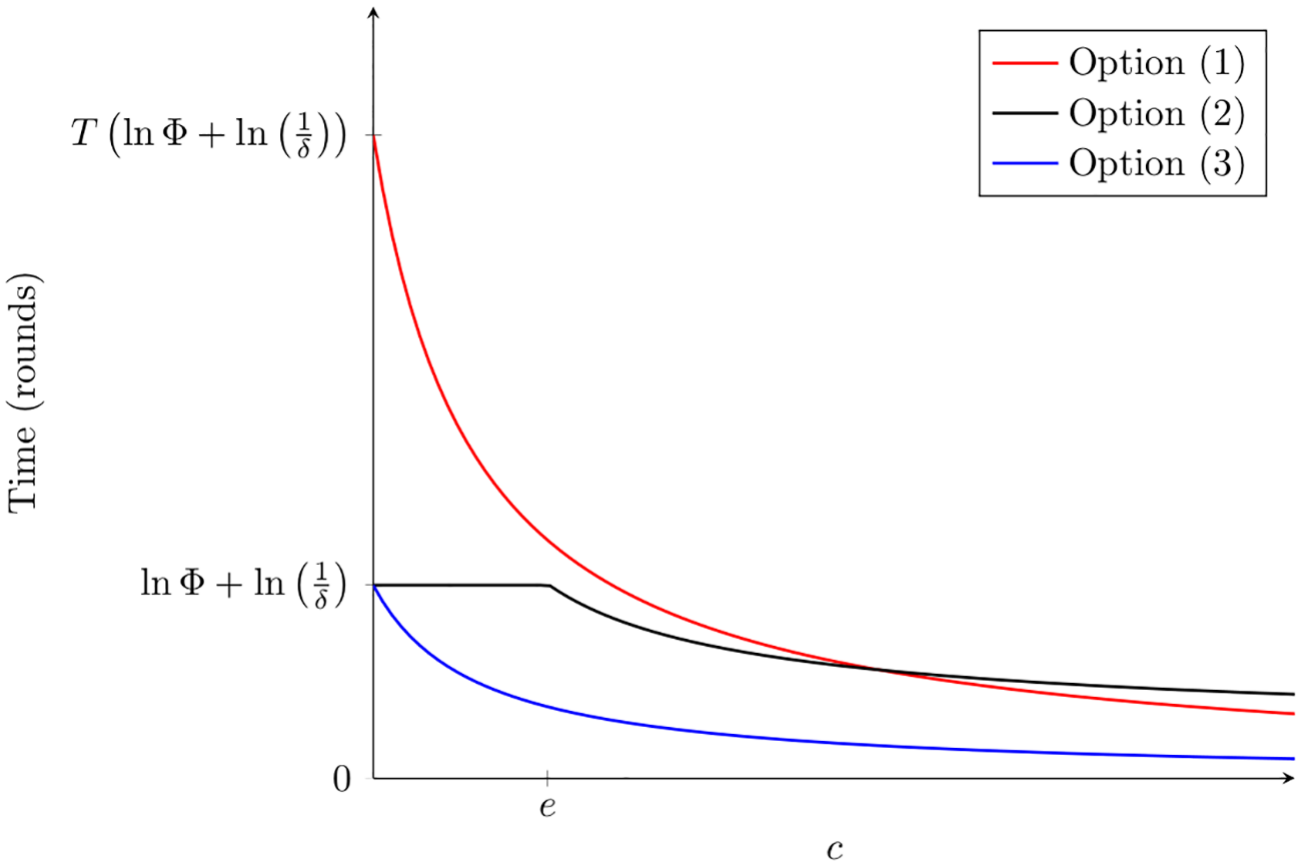
Time for workers to re-allocate as a function of *c*. The three plots indicate the times until workers re-allocate successfully for options (1), (2), and (3) of the *chocie* component as a function of *c*. The *x*-axis denotes the value of *c*, and the *y* axis denotes the time for workers to re-allocate. For options (1) and (3) the plotted function is approximately 1/*c* multiplied by the corresponding time to re-allocate for *c* = 1. For option (2), the plotted function is approximately 1/ln *c*, truncated at the time for workers to re-allocate for *c* = 1.

First, we consider the case where the demand *D* has to be fully satisfied with a high probability (1 − *δ*). For this case, in options (2) and (3), we see that if the number of task types (|*T*|) is small, the time to allocation only depends on this parameter (see also Table 4). If the number of task types is high, we see a positive (logarithmic) dependence of the time to correctly re-allocate all workers on the deficit across all tasks (i.e. the value of Φ). That is, correct allocation takes longer if more workers have to be re-allocated; this relationship is not linear but saturates over time. In the case of option (1) (where workers can only check for demand in different tasks sequentially rather than instantaneously), we also see a linear positive dependence on the number of tasks |*T*|. Finally, as the workers-to-work-ratio (*c*) increases, the time to re-allocate all workers decreases: this means that if there are ‘extra workers’ (workers in excess of the total demand for work), task allocation becomes faster. In options (1) and (3), that dependence is approximately 1/*c*, and in option (2), the dependence is slightly weaker: 1/ln *c* (Fig 1). However, note that extra ants do not contribute towards a faster task allocation until *c* is large enough (approximately until *c* ≥ *e*).

**Table 4:** Numerical results.

Insect name	|*T*|	*c*	Φ	1 − *δ*	1 − *ϵ*	(1)	(2)	(3)
Honey bee (Apis mellifera) predator attack	10	1.3	5000	0.95	0.7	708.49 (258.44)	10 (10)	6.32 (4.73)
Honey bee (Apis mellifera) change in foraging conditions	10	1.3	150	0.8	0.7	407.39 (173.13)	10 (10)	4.93 (3.35)
Rock ants (Temnothorax rugutulus) change in foraging conditions	4	1.7	5	0.5	0.7	43.34 (35.71)	4 (4)	2.69 (2.43)
Rock ants (Temnothorax rugutulus) emigration after nest breakdown	4	1.7	25	0.9	0.9	103.93 (86.69)	4 (4)	4 (4)
Bumble bee (Bombus impatiens)	8	1.5	5	0.9	0.75	166.91 (157.39)	8 (8)	4.62 (4.3)

We calculated the time to successful allocation, in the three options of our model, using numerical parameter values that approximate the conditions in some example cases of task re-allocation in social insects. For each option, we calculate the number of rounds until the entire demand *D* (consequently, the entire initial deficit Φ) is satisfied and, in parentheses, the number of rounds until a (1 − *ϵ*) ⋅ Φ fraction of the demand is satisfied. These are not intended to be exact time estimates; the values for *c*, *δ*, and *ϵ* have not been estimated empirically for any species, nor is it clear how long a ‘round’ precisely should be. The intent, here, is to check whether task allocation might take a significant amount of time in realistic scenarios (and thus be considered a difficult problem, and its solutions, i.e. task allocation algorithms, subject to natural selection). These numerical estimates also serve to illustrate how the different parameters affect the time to successful reallocation in a realistic context of other parameter values.

Second, we studied the time until the demand *D* in different tasks is satisfied approximately (to within a (1 − *ϵ*) fraction) rather than exactly as above (but still with high probability of 1 − *δ*). In general, the effect of different parameters on performance is similar to the case where task demands are satisfied exactly. However, we show that in this case, for all options of *choice*, surprisingly, the time to re-allocate all workers does not depend on the total deficit (Φ) at all. Instead, it depends on the value of *ϵ*. In particular, the smaller *ϵ* gets, the more accurately we need to re-allocate all workers, leading to a longer time to do so, until the same time as for the exact case is reached (as in the first row in Table 4).

The results in both cases (exact and approximate matching of task demands) are the same for *ϵ* = 1/Φ. This implies that for very large Φ, *ϵ* needs to be very small in order to have equal values in the two rows. Approximate task allocation is achieved faster than precisely accurate task allocation when Φ > 1/*ϵ*.

Finally, for the third option of the *choice* component, we also study the time to re-allocate all workers under some noise in the *success* and *choice* components. In particular, we assume the *success* component can make a limited number of ‘mistakes’ (at most *z* flipped bits from 0 to 1 and vice versa) and the *choice* component may return a task with a probability slightly larger or smaller than we require in option (3) (change the probability of a task being suggested to a worker by at most a factor of 1 − *y*). We show that the best the workers can do in re-allocating is to satisfy all but *z* units of work, and the time to reach such a re-allocation increases as the range of the probabilities of *choice* increases.

### Numerical results

**Fig 2:**
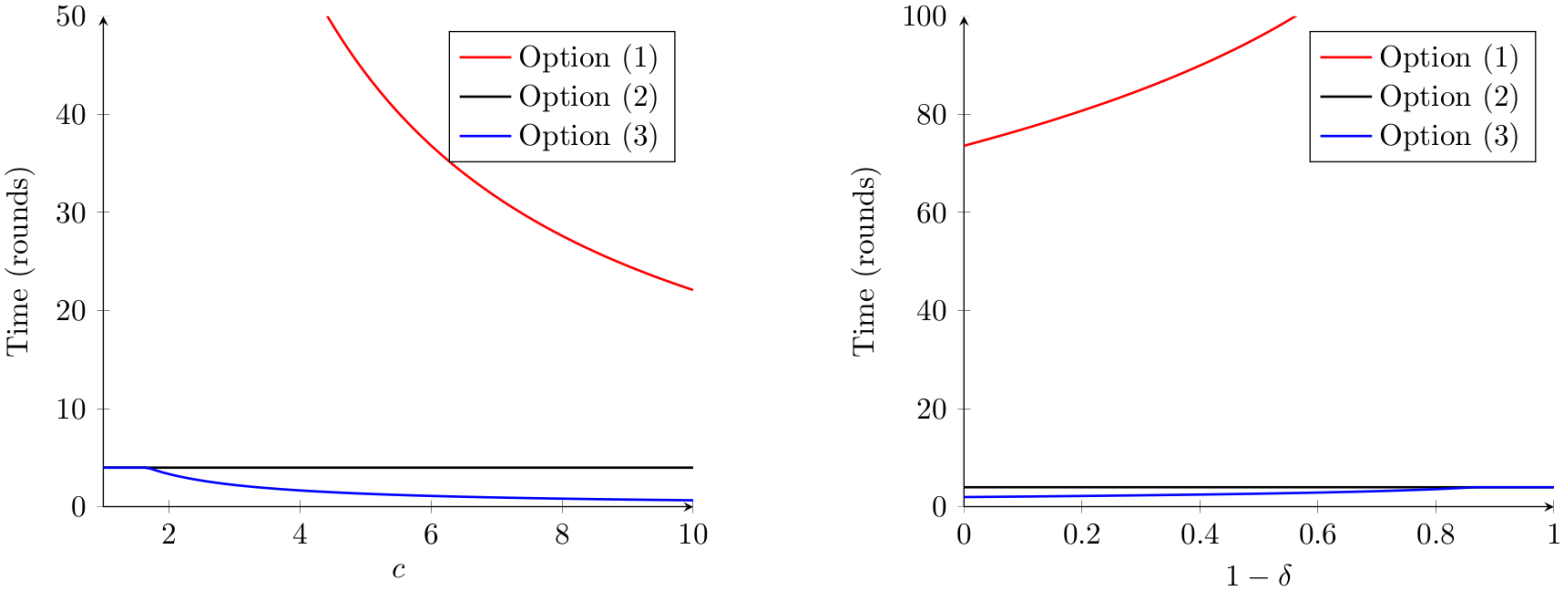
Time for workers to allocate as a function of *c* and 1 − *δ*. The two plots indicate the times until workers re-allocate successfully for options (1), (2), and (3) of the *chocie* component as a function of *c* and 1 − *δ* respectively, with specific parameter values assumed (compare the left plot to Fig 1). For both plots, we assume |*T*| = 4, Φ = 10, and *ϵ* = 0. Additionally, for the plot on the left, we assume 1 − *δ* = 0.99, and for the plot on the right, we assume *c* = 1. For the plot on the left, the y-intercept for option (1) (corresponding to *c* = 1) is approximately 221 (and thus this is also the value for option 1 at 1 − *δ* = 0.99 in the right plot.

Here, we choose some sample values for the parameters in the model and calculate numerical results (Table 4 and Fig 2). The expressions used to generate these values roughly correspond to the first two rows of the table in Table 3, with the difference that here the values are exact upper bounds and not asymptotic (big-oh) notation (see S2 Text for how they are calculated).

The most obvious pattern here is that task allocation takes a lot more rounds under option (1) (workers are not able to assess quickly which tasks need more work) than under options (2) and (3) for choice. Is task allocation then a ‘difficult’ problem that requires a significant amount of time? This depends on how long, in real time, a ‘round’ is. If workers require time on the order of minutes to choose a task, attempt to perform work in it, and assess whether they have successfully contributed to the colony with this work, then the results for option (1) imply that a colony will need one or several hours to correctly match workers to tasks when the demand for work in the different tasks changes. For the examples given here, that would imply a definite cost, in terms of not being able to maintain a correct match of workers to the tasks that need work (since the level of demand for work is likely to change more frequently than every few hours, or because a lag in matching demand in the realm of hours implies a significant cost). If workers only require seconds to assess demand for work across all tasks (e.g. because task stimuli are volatile pheromones, or global variables like temperature), and can choose a task based on this information, then the time cost of correct allocation in options (2) and (3) is likely insignificant. This would imply that a correct allocation can be achieved quickly, and thus workers should be dynamically and optimally reallocated to changing demands on a timescale of less than a minute.

Another pattern emerging from these calculations is that under options (2) and (3) for choice, it is primarily the number of task types (|*T*|) that affects how fast task allocation proceeds. Neither the number of extra workers (*c*) nor the size of the initial work deficit (Φ) play a major role; also neither does *ϵ*, i.e. allowing a small amount of error in allocation does not decrease the time to successful reallocation in a meaningful way. How accurate are these conclusions, given that we are only examining somewhat arbitrarily chosen parameter combinations? Our results in Table 3 give a more complete picture, as do the plots in Fig 2; this table is only intended as an illustration of the results. However, the parameter values illustrated here are not entirely arbitrary, but represent best-guesses given empirical data (see Table 4). For example, many authors have tried to examine the number of task types in social insects, and our results cover the range generally found (2–30; Table 4).

## Discussion

Modeling, in general, can serve different purposes in the scientific process [62, 63]. Our paper has the goal of examining, first, whether ‘task allocation’, i.e. the process of using a distributed, self-organized algorithm to dynamically match workers to work, is a difficult problem, and second, what factors determine the optimal algorithm to achieve such task allocation. Our paper thus aims to provide a ‘proof of principle’ sensu [63]: namely, we aim to show under which factors should be expected, or not expected, to affect the performance of task allocation mechanisms given certain assumptions. Next, we survey the relevant work on theoretical modeling and empirical studies of task allocation; then, we discuss our results, and examine the assumptions we made in the model to achieve them.

### Related work

The process of task allocation and its typical outcome, division of labor, have received a lot of attention in the social insect literature. Empirical studies typically focus on determining the individual traits or experiences that shape, or at least correlate with, individual task specialization: e.g. when larger or older individuals are more likely to forage (e.g. [53]) or when interaction rates or positive experience in performing a task affect task choices [32, 64]. Generally the re-allocation of workers to tasks after changes in the demand for work often needs to happen on a time scale that is shorter than the production of new workers (which, in bees or ants, takes weeks or months, [65]), and indeed empirical studies have found that the traits of new workers do not seem to be modulated by colonies to match the need for work in particular tasks [66]. Therefore, more recent empirical and most modeling studies focus on finding simple, local behavior rules that generate individual task specialization (i.e. result in division of labor at the colony level), while simultaneously also enabling group-level responsiveness to the changing needs for work in different tasks [35, 67, 68]. For example, in classic papers, Bonabeau et al. [69] showed theoretically that differing task stimulus response thresholds among workers enable both task specialization and a flexible group-level response to changing task needs; and Tofts and others [70, 71] showed that if workers inhabit mutually-avoiding spatial fidelity zones, and tasks are spread over a work surface, this also enables both task specialization and flexible response to changing needs for work.

In this paper we examined how well we should expect task allocation to be able to match actual demands for work, and how this will depend on group size and the number of ‘extra’, thus inactive, workers. Neither of the modeling studies cited above explicitly considered whether task allocation is improved or hindered by colony size and inactive workers. In addition, while several studies find increasing levels of individual specialization in larger groups, the empirical literature overall does not show a consensus on how task allocation or the proportion of inactive workers is or should be affected by group size (reviewed in [14, 22]).

In general, few studies have cosidered the efficiency of the task allocation process itself, and how it relates to the algorithm employed [72], often in the context of comparing bio-(ant-)inspired algorithms to algorithms of an entirely different nature [73, 74]. For example, Pereira and Gordon, assuming task allocation by social interactions, demonstrate that speed and accuracy of task allocation may trade off against each other, mediated by group size, and thus ‘optimal’ allocation of workers to tasks is not achieved [72]. Duarte et al. also find that task allocation by response thresholds does not achieve optimal allocation, and they also find no effect of colony size on task allocation performance [75]. Some papers on task allocation in social insects do not examine how group size *per se* influences task allocation, but look at factors such as the potential for selfish worker motives [76], which may be affected by group size, and which imply that the task allocation algorithm is not shaped by what maximizes collective outcomes. When interpreting modeling studies on task allocation, it is also important to consider whether the number of inactive workers is an outcome emerging from particular studied task allocation mechanisms, or whether it is an assumption put into the model to study its effect on efficiency of task allocation. In our study, we examined how an assumed level of ‘superfluous’, thus by definition ‘inactive’, workers would affect the efficiency of re-allocating workers to tasks after demands had changed.

While the above models concern the general situation of several tasks, such as building, guarding, and brood care, being performed in parallel but independently of one another, several published models of task allocation specifically consider the case of task partitioning [77], defined in the social insect literature as a situation where, in an assembly-line fashion, products of one task have to be directly passed to workers in the next task, such that a tight integration of the activity in different tasks is required. This is, for example, the case in wasp nest building, where water and pulp are collected by different foragers, these then have to be handed to a construction worker (who mixes the materials and applies them to the nest). Very limited buffering is possible because the materials are not stored externally to the workers, and a construction worker cannot proceed with its task until it receives a packet of water and pulp. One would expect different, better-coordinated mechanisms of task allocation to be at work in this case. In task partitioning situations, a higher level of noise (variation in availability of materials, or in worker success at procuring them) increases the optimal task switching rate as well as the number of inactive workers, although this might reverse at very high noise levels [78]. Generally larger groups are expected to experience relatively lower levels of noise [79]. In this line of reasoning, inactive workers are seen as serving a function as ‘buffer’ (or ‘common stomach’, as they can hold materials awaiting work) [79, 80]; this implies that as noise or task switching rate increase, so does the benefit (and optimal number) of inactive workers.

### Does task allocation matter?

Is task allocation a difficult problem, and does it matter which algorithm is chosen? If task allocation is an easy problem, then the match of work to workers can be achieved without significant costs. If task allocation is difficult, on the other hand, the choice of task allocation algorithm matters for system performance; in biological systems where this is the case, we would expect task allocation mechanisms to be under strong selection, and their evolution to reflect the specific ecological context of the system. In social insect colonies, for example, task allocation mechanisms appear to differ between species—this could be the case because they are not under selection, and different species happen to have hit on different, equally good, solutions, or because they are under selection, and different species have different requirements (e.g. because they differ in the frequency with which demand for work in different tasks changes). There is some evidence that even brief mismatches of work to workers, i.e. incorrect task allocation, can be detrimental in certain species (e.g. because brood do not develop well when briefly not thermoregulated [81]).

Here we estimate the time to correct allocation for several species and contexts (Table 4). For example, we estimate that when a honey bee colony is attacked by a large predator, and 5000 (±30%) bees should ideally be allocated to defense, the time to achieve this within our generalized task allocation algorithm would be around 5 − 10 rounds if all bees can directly sense the need for more defenders (options (2) or (3)), and 700 rounds if they cannot (and only arrive in the defense task because they randomly tested different tasks in different rounds, option (1)). Since this particular situation requires a quick collective response, the difference between option (1) and options (2) or (3) appears meaningful, regardless of whether a ‘round’ takes minutes or seconds to complete. In another example, a change in foraging conditions in the case of rock ants (Temnothorax) may imply that only five additional workers need to be allocated to the task of foraging; however, in that system it appears likely that individuals need on the order of a minute rather than seconds to assess both the state of their environment and whether their own task performance is successful (in the sense of fulfilling a demand). If that is the case, a delay of 40 rounds may also be a meaningful and costly delay to appropriately exploiting novel food sources, for example. In all cases, the main effect on the difficulty of task allocation is whether or not individuals can assess the demand across different tasks simultaneously (instead of only in the one task they are working on), and what time period a ‘round’ in our model corresponds to (i.e. how long it takes a worker to assess whether its current work is needed, i.e. whether it is ‘successful’ in the task according to the terms used in our model). In addition, the costs as presented in Table 4 have to be paid each time the demands for work in different tasks change, and workers have to be reallocated to match these new demands. Overall, our calculations show that realistic parameter estimates can lead to potentially meaningful costs of slow task allocation. Our calculations are pretty coarse however, as the precise values of many of the parameters are not known (however see Table 2 for references on parameter estimates). More empirical work in this area would be useful.

Our work also addresses a more general question. Division of labor is widespread in complex systems from developing embryos to human organizations; it implies a degree of individual specialization, i.e. more or less consistent differences between individuals in the tasks chosen. Division of labor is often associated with ‘progress’ or ‘increase in complexity’ (e.g. [17]). All systems with division of labor must implement some algorithm that lets individuals choose their task. How do these task allocation algorithms evolve, i.e. which external or internal conditions select for which kinds of mechanism? For example, under which conditions and in which systems do the best task allocation algorithms produce highly specialized workers, insensitive to small changes in demands across tasks? One might argue that in a system with highly specialized workers, the cost of allocation mismatch is never more than the average allocation minus current demands, because the system can make specialized workers in the correct proportion for the average expected allocation. Any algorithm that allows workers to be fully generalist, i.e. to freely switch between any tasks, must ensure that the mismatch of workers to demands is not on average greater than that. Understanding more about why particular task allocation mechanisms are selected for would thus increase our understanding about the evolution of specialization and division of labor more generally.

### Colony size does not affect ease of task allocation

Does colony size lead to a change in which task allocation algorithms perform well, and does it lead to selection for specialization? The answers to these questions are not straightforward (and neither are the empirical results on this [22]). Contrary perhaps to conventional wisdom in both biology and computer science, we do not find a direct dependence of the time to solve the task allocation problem on ‘colony size’ or ‘problem size’, if we assume that the total amount of work scales linearly with the number of workers (*c* = |*A*|/*D*, the number of workers per work available, is constant across different |*A*|). This holds even if all work only has to be satisfied with a certain probability, and if only close to the total needed work has to be satisfied. This result is perhaps logical because we implemented neither the type of noise that would lead to a benefit of large numbers (where the relative amount of variation in environments experienced decreases with colony size), nor did we implement any economies of scale (there are no broadcast signals; we did not model any communication explicitly, and if the task feedback is thought of as the result of communication, we did not implement any constant costs with colony size). No matter how logical in hindsight however, this was not what we had intuitively expected nor what is sometimes suggested in the literature [22].

If we find empirically that in some systems the level of specialization or the task allocation mechanism implemented change with colony size, some factors not modeled here have to be at play: e.g. fixed costs leading to economies of scale, or non-linear scaling in the effectiveness of communication. For example, it may be that the feedback on whether an individual worker contributes to reducing a deficit depends on social interactions that do not scale linearly with colony size. This is plausible of course (and has been demonstrated empirically in some cases, e.g. [50]). Importantly however, it is not obvious that task allocation will perform better at larger colony sizes in all systems. It is worth noting that even if the time to correct allocation did change with colony size, this does not make obvious predictions for the evolution of division of labor (the degree to which workers should be specialized). If task allocation is difficult (takes a long time), it may be that colonies abandon the attempt to dynamically reallocate workers at all, and instead employ specialized, ‘preprogrammed’ workers in proportions of the average expected demands across tasks.

### The amount of work available per worker affects ease of task allocation

We discover that to understand the dependence of task allocation on the number of workers in the colony (|*A*|), actually what we really need to know is (*D*), the total amount of work that needs to be done. Note that *D* refers to currently open tasks, thus is not likely to be ‘unlimited’; in social insects, if nothing else, in the short term, available work will be limited by the queen’s egg laying rate. This total amount of work available (or necessary) has not been studied explicitly either empirically or in models of social insect task allocation, with few exceptions [28]. So, we do not have a good understanding of how *D* behaves with |*A*| intra- or inter-specifically. Here we have simply assumed that |*A*|/*D* is constant, but this may well not generally be so: previous studies and conceptual papers have suggested either that larger colonies are relatively less productive, perhaps suggesting that less work is available per worker, or that they are more productive (because they are capitalizing on some economies of scale) — it is unclear what the latter would imply for the amount of work per worker available. One interesting new hypothesis here is that the evolution of task allocation across social insects may, in part, be driven by the factors that limit productivity -– e.g. is the colony raising brood at near the queen’s maximal egg laying rate? In this case *D* may increase less than linearly with increasing colony size, and thus task allocation may become easier, even trivial, at higher colony sizes. Our modeling study thus suggests a new hypothesis (one for the purposes of modeling more generally, [82]), by providing the insight that a previously ignored variable impacts the outcome of a well-studied process.

### ‘Extra’ workers make task allocation faster

Our results also suggest that *c* (the ratio of |*A*|/*D*, or the number of workers divided by the amount of work available) matters, and higher *c* generally leads to faster allocation time. Thus colonies may benefit from having more workers available than work. This is a novel hypothesis for the existence of ‘inactive’ workers in social insect colonies and other complex systems [14]. That is, colonies may produce more workers than needed to complete available work simply in order to speed up the process of (re-)allocating workers to work, and thus potentially reducing costs of temporary mismatches of workers with needed work. In other words, inactive, ‘surplus’ workers in colonies may increase colony flexibility and how close colonies get to an ‘optimal’ task allocation in environments where task demands often change and workers frequently have to be reallocated. The benefit of extra workers (higher *c*) does not depend on colony size (|*A*|), thus we would expect both large and small colonies to have as many extra workers as they can afford. Although the dependence on *c* varies with task allocation algorithm (it is least strong in option (2)), higher *c* is always beneficial.

Apparently inactive workers are common in social insect colonies. While these workers may be selfish [40, 41] or immature [42], or temporarily unemployed due to fluctuating total demand [14], our work here thus implies that they may also be present simply to improve task allocation. That is, colonies may produce extra workers such that some workers are ‘unemployed’ at all times on average, but so that the time to correct reallocation of workers when demands across tasks change is minimal. This is a novel hypothesis for the function of inactive workers in complex systems more generally.

### The number of task types matters

It is intuitive that task allocation may be more difficult if workers have to choose among many different possible tasks to perform (high |*T*|). However, we show that the effects of |*T*| are mixed and depend both on the information available to workers and the actual combination of parameter values, particularly on the size of |*T*|. Specifically, in the parameter ranges we explored numerically (based on empirically plausible parameter values), the time to correctly allocated workers to tasks depends linearly on the number of task types for options (1) and (2), and not at all for option (3). In option (1), where workers effectively have to ‘test’ tasks sequentially to discover where work is needed (because they only find out through the *success* mechanism), |*T*| always enters into performance as a linear factor. This would be the case for example if workers have to walk to different locations in the nest, or if they have to invest some other significant effort into assessing demand for each specific task. In options (2) and (3), workers can effectively assess demand across all tasks in parallel; this may be the case if task demand is communicated through global stimuli, such as temperature or volatile pheromone levels. In such a case, the number of task types matters only if it is lower than the second term in the minimum function (for example, see Corollary C.6 in S2 Text). Thus, whether the number of task types affects task allocation performance depends on the context of other parameter values.

What do we know about |*T*| empirically? Several authors have attempted to quantify this number (see Table 2). However, empirically studies have often acknowledged that what are ‘separate tasks’ and what are just elements of the same task is difficult to define, and that this may lead to number estimates that are quite subjective. In our model, workers within the same task are assumed to immediately (within one round) correctly distribute the work among themselves, whereas the demand for work in a different task is only assessed via the *choice* and *success* feedback mechanisms as defined above. So, one may think, for example, of each item to be worked on as a ‘task’ (e.g. each larva that needs tending and feeding, or each breach in the wall), in which case |*T*| might be a quite large number; or one may think that all larvae are part of the single task of brood care, and all places in the wall that need repair are part of the task of nest building, in which case |*T*| is likely to be a small number (perhaps below 20, or even below 10). Which is the more appropriate way of counting tasks, in the context of our model, depends on whether, for example, each ant worker dedicated to brood care will be able to immediately assess which particular larvae need care, not loosing time in arriving at a consensus with other brood care workers about who is tending to which exact brood item, or alternatively where each brood care worker can jointly and concurrently contribute to the work in that task without internal coordination required at the timescale of overall task allocation.

### Assumptions made in our approach

The results presented in this paper were derived using methods from the field of theoretical distributed computing. The problems considered in this field are very similar to those that are relevant in the biological study of distributed systems—and almost all biological units, from cells with their metabolic and molecular networks to ecosystems, are really distributed systems. We believe that the techniques and results from theoretical distributed computing may lead to many novel approaches and insights in biology in the future, and interdisciplinary work in this area is increasing [29, 46, 47, 83, 84]. In particular, research in theoretical distributed computing has examined the limitations of distributed algorithms, for example in such contexts as distributed task allocation as we study here.

Generally, this field analytically derives results about models that often assume stochastic individual behavior, and in particular quantifies system-level performance given specific individual algorithms (i.e. behavioral rules). Here, we have analyzed how our model, a generalized form of an insect-inspired task allocation algorithm, performs in terms of how long it takes to correctly allocate workers to different task types which need work. We have allowed for approximate solutions, by looking at the time to allocating workers correctly only with a certain minimum probability (1 − *δ*), and only to within *ϵ* of the best allocation. We have also allowed for errors in the demand assessment function, e.g. if workers make mistakes when assessing whether they are needed in a particular task. We have made the assumption that the relevant measure of how well a task allocation mechanism performs is related to the time to correct allocation, that is the time until workers are matched to tasks that need work. Other performance measures are possible, such as assessing how quickly the task-worker match approaches an ideal allocation, or how good the match can ever get; or entirely different parameters may be under selection, such as how much workers have to switch tasks [38], how well workers prioritize more important tasks over unimportant ones, or how much information workers need to collect in order to allocate correctly.

Second, our approach makes another assumption about how the performance of a task allocation mechanism is measured: we only quantify this performance for the worst-case inputs, namely the configuration of task deficits (i.e. the distribution of unfulfilled demands across tasks) that leads to the longest possible time to re-allocate. Thus, while stochasticity in worker decisions and information is taken into account and expected results derived, we do not make any assumptions about what configuration of task deficits workers are likely to encounter. If this was known, more precise expectations for performance could be derived. In distributed computing theory, there is a general assumption that such a worst-case scenario (generally called the upper bound of performance) is a good measure of algorithm performance; however it does not need to be close to the overall expected case.

Finally, we make the crucial assumption that all workers are identical in preferences and skills. Thus, our model represents a system of flexible, homogeneous workers. If workers randomly differed in their ability to perform different tasks, matching them optimally to tasks with changing demands for work becomes an extremely hard problem [12]. On the other hand, worker skills in a task may be linked to their preferences for that task, either because these are innately linked, or because workers learn to prefer the tasks they do well, or learn to do the tasks well they prefer [85]. How much the dynamic (re-)allocation of workers in response to changing demands in different tasks is affected by such worker heterogeneity remains to be analyzed.

### Conclusion

We mathematically derived how the time it takes to correctly allocate workers to work is affected by several factors, such as colony size and the number of ‘extra’ workers. We make only minimal assumptions about the algorithm used, and we explore several ways of measuring performance of task allocation, which means these relationships should hold fairly generally. Our model brings several insights. First, costs or benefits of group size do not arise in task allocation ‘automatically’, that is from minimal assumptions such as ours. Second, such a result clarifies our thinking and suggests how, for example, colony-size-dependencies may occur (e.g. if information on work deficits is communicated faster in larger colonies), thus guiding future research as well as identifying which variables qualitatively affect system behavior. One such variable is the amount of work available; this has not been considered in previous empirical studies but appears to be a crucial factor affecting the evolution of task allocation algorithms [28]. Third, the model results have generated a novel hypothesis for the existence of inactive workers in social insect colonies [14], namely that they may serve to speed up the task allocation process. It now can be studied whether this may be the reason for their evolution. All of these results are derived analytically, using approaches from theoretical distributed computing, without the need for parameter estimation such as would be necessary in a simulation study. In summary, our ‘proof of concept’ model sensu [63] helps to identify how limitations and processes at the individual level can affect group level processes in a distributed system.

## Supporting information

S1 Text Formal definitions. We provide mathematically rigorous definitions of our task allocation model.
(PDF)

S2 Text Full proofs. We provide full formal proofs of the mathematical statements in the Results section.
(PDF)
