# Supplementary material for: Costs of task allocation with local feedback: Effects of colony size and extra workers in social insects and other multi-agent systems
Source: PLoS Comput Biol. 2017 Dec 14;13(12):e1005904. doi: 10.1371/journal.pcbi.1005904 (PMC5746283; doi:10.1371/journal.pcbi.1005904)
Supplement: S1 Text — We provide mathematically rigorous definitions of our task allocation model. (PDF) [file pcbi.1005904.s001.pdf]

Costs of Task Allocation with Local Feedback: Effects of Colony Size and Extra Workers
(S1 Appendix)

Tsvetomira Radeva, Anna Dornhaus, Nancy Lynch, Radhika Nagpal, Hsin-Hao Su

Formal Definitions and Problem Statement

Let A denote the set of workers and T denote the set of tasks. Each task $i \in T$ has an integer demand d_i that represents the minimum number of workers required to work on task i in order to satisfy the task. Let w_i denote the total number of worker units of work currently supplied to task i. Let $\vec{w}$ and $\vec{d}$ denote the vectors of w_i and d_i values, respectively, for each $1 \leq i \leq |T|$. The $\vec{d}$ vector is static, while $\vec{w}$ changes over time depending on the different tasks workers choose to work on.

Clearly, in order for all demands to be met, there should be sufficiently many workers in the colony. We assume that there exists a real $c \geq 1$ such that $|A| = c \cdot \sum_{i \in T} d_i$.

The structure of the task allocation system we consider is illustrated in Figure 1. The environment component contains state information about all tasks, their demands and the work supplied to each task at the current point in time. The environment component informs the *success* and *choice* feedback components of the demands and the work provided to tasks. The *success* component provides each worker component with information $success(b), b \in \{0, 1\}$ about the success of the worker at the task i it is currently working on. The *choice* component provides each worker component with information $choice(i), i \in T \cup \{\bot\}$ about some (alternative) task i, where $\bot$ indicates "no task". Finally, each worker component updates the environment with the work it has completed on task $i \in T \cup \{\bot\}$ through $work(i)$. A worker component output $work(\bot)$ indicates that the worker did not complete any work.

Environment: The environment consists of the set of tasks, their demands and the number of workers currently working on each task. The environment outputs the $\vec{d}$ and $\vec{w}$ vectors to the *success* and *choice* components, providing them with information about the work supplied to tasks and their demands. The input to the environment component is the work that each worker provides to each task denoted by $work(i), i \in T \cup \{\bot\}$. As a result of this input action, the environment updates the $\vec{w}$ vector accordingly.

Workers : Each worker $a \in A$ has a state $q \in Q = \{q_\bot, q_1, q_2, \cdots, q_{|T|}\}$ at each point in time, where $q_\bot$ indicates that worker a is not working on any task and each state q_i, for $i \in \{1, \cdots, |T|\}$, indicates that worker a is working on task i. Each worker is modeled as a finite state machine with transition function $\delta : Q \times (\{0, 1\} \times (T \cup \{\bot\})) \rightarrow Q$; in other words, each worker's new state is determined by its old state and its inputs from the *success* and *choice* components. Let q be the current state of some worker a, and let q' be the resulting state of worker a after applying δ. In

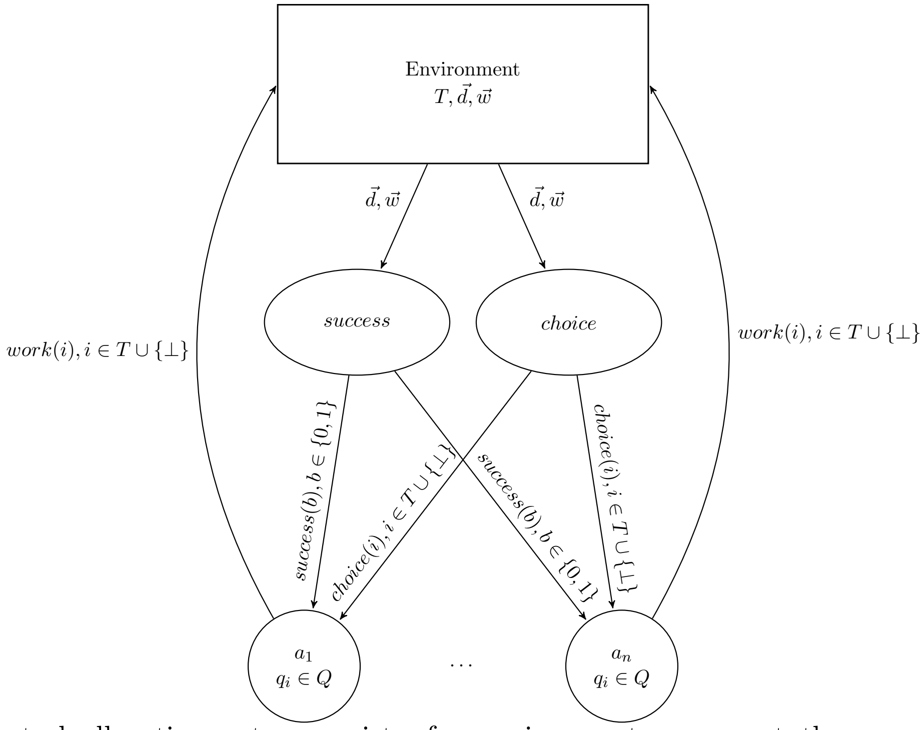

Figure 1: The task allocation system consists of an environment component, the *success* and *choice* feedback components, and n worker components.

each step, q' is determined as follows: $q' = q$ if *success* outputs 1, and $q' = q_i$ if *success* outputs 0 and *choice* outputs $i \in T \cup \{\bot\}$. The new state q' of the worker directly determines its output to the environment component.

Feedback: The environment feedback components *success* and *choice* provide each worker with a boolean and a task in $T \cup \{\bot\}$, determined based on $\vec{w}$ and $\vec{d}$. The input to the feedback components is the $\vec{d}$ and $\vec{w}$ vectors. The output of *success* is a boolean $\{0, 1\}$, and the output of *choice* is some task in $T \cup \{\bot\}$. Since randomness usually plays a role in the environment, the output values of *success* and *choice* may be determined randomly.

Execution: The execution of any algorithm solving the task allocation problem starts at time 0 and proceeds in synchronous rounds, such that each round $r + 1$, for $r \geq 0$, denotes the transition from time r to time $r + 1$. In each round $r + 1$, the environment component provides outputs to *success* and *choice* containing the demand and work vectors $\vec{d}$ and $\vec{w(r)}$. The *success* and *choice* components provide each worker component with a boolean ($success(b), b \in \{0, 1\}$) and a task ($choice(i), i \in T \cup \{\bot\}$) output. Each worker component performs a state transition using its δ transition function and updates the environment component with the work ($work(i), i \in T \cup \{\bot\}$) it performed. An execution α is a sequence of alternating (1) states of the environment, and mappings from workers to (2) *success* outputs, (3) *choice* outputs, and (4) work inputs. Formally, $\alpha = (Q_0, S_1, C_1, W_1, Q_1, S_2, C_2, W_2, \cdots)$, where, for each $r \geq 0$, Q_r is the state of the environment at time r, S_{r+1} is a mapping of type $A \to \{0, 1\}$, C_{r+1} is a mapping of type $A \to T \cup \{\bot\}$, and W_{r+1} is a mapping of type $A \to T \cup \{\bot\}$. The S_{r+1} mapping refers to the *success* outputs to all

workers in round $r+1$, the C_{r+1} mapping refers to the *choice* outputs to all workers in round $r+1$, and W_{r+1} mapping refers to the inputs to the environment from all workers in round $r+1$.

Problem Statement: A state s_r of the environment component at time $r \geq 0$ *satisfies* some task $i \in T$ if $d_i \leq w_i(r)$. An execution α satisfies all tasks if there exists a time $r \geq 0$ such that for each $r' \geq r$, state $s_{r'}$ of the environment satisfies task i for all $i \in T$. A probabilistic execution α satisfies all tasks with probability $1-\delta$, for any $0 < \delta < 1$, if, with probability at least $1-\delta$, there exists a time $r \geq 0$ such that for each $r' \geq r$, state $s_{r'}$ of the environment satisfies task i for all $i \in T$.

The specification of *success* and some of the specifications of *choice* in this section are inspired by the biological model by Pacala et al. [1] and simplified for the sake of easier analysis.

Success Component The first component, *success*, determines whether each worker is successful at the task it is currently working on. Throughout this paper, we consider *success* components that satisfy the following conditions in each execution and at each time r of the execution: for each task $i \in T$, $|\{a \mid a \text{ is in state } q_i \text{ at time } r \text{ and receives } \textit{success}(1) \text{ in round } r+1\}| = \min(d_i, w_i(r))$. Also, each worker in state $q_\perp$ at time r receives *success*(0) in round $r+1$. The *success* component provides information that allows excess workers working on a satisfied task to switch to another task.

Choice Component The *choice* component returns a candidate task to each worker as an alternative task to work on. We consider five different specifications of *choice*:

1. *choice* returns a task drawn from all the tasks in T uniformly at random.
2. *choice* returns a task drawn from the set of unsatisfied tasks, $U(r) = \{i \mid d_i > w_i(r)\}$, uniformly at random. If there is no such task, then *choice* returns $\perp$.
3. *choice* returns a task i drawn from the set of all unsatisfied tasks with probability $(d_i - w_i(r))/\sum_{j \in U(r)}(d_j - w_j(r))$. This option corresponds to the scenario where workers can somehow sense the need to work on each task, and are more likely to work on tasks with high deficit $d_i - w_i(r)$ compared to the total deficit of all unsatisfied tasks $\sum_{j \in U(r)}(d_j - w_j(r))$.

References

[1] S. W. Pacala, D. M. Gordon, and H. C. J. Godfray. Effects of social group size on information transfer and task allocation. *Evolutionary Ecology*, 10:127–165, 1996.
