# Supplementary material for: Costs of task allocation with local feedback: Effects of colony size and extra workers in social insects and other multi-agent systems
Source: PLoS Comput Biol. 2017 Dec 14;13(12):e1005904. doi: 10.1371/journal.pcbi.1005904 (PMC5746283; doi:10.1371/journal.pcbi.1005904)
Supplement: S2 Text — We provide full formal proofs of the mathematical statements in the Results section. (PDF) [file pcbi.1005904.s002.pdf]

Costs of Task Allocation with Local Feedback: Effects of Colony Size and Extra Workers
(S2 Appendix)

Tsvetomira Radeva, Anna Dornhaus, Nancy Lynch, Radhika Nagpal, Hsin-Hao Su

A General Definitions and Lemmas

In this section, we give some basic definitions and results that will be used in the subsequent analyses of the convergence times for the various *choice* options.

Definitions. All definitions are with respect to a fixed execution α. For each task $i \in T \cup \{\perp\}$ and each time r, let $A_i(r)$ denote the set of ants in state q_i at time r. A task is *satisfied* at time r if $d_i \leq w_i(r)$. Let $S(r)$ denote the set of satisfied tasks at time r. Let $U(r) = T \setminus S(r)$ denote the set of unsatisfied tasks at time r.

We begin by showing some basic properties of the *success* component. These results hold regardless of the particular *choice* component specification. Consider an arbitrary execution α of the task allocation system with the *success* component and any one of the three *choice* components.

Lemma A.1. *For each task $i \in T$, each time r, each time r', such that $r' \geq r$, and each $d \in \mathbb{N}$, such that $d \leq d_i$, if $w_i(r) \geq d$, then $w_i(r') \geq d$.*

Proof. Consider fixed task $i \in T$, time r, and value $d \leq d_i$, and suppose that $w_i(r) \geq d$. The proof is by induction on r' for $r' \geq r$. In the base case $r' = r$, and by assumption $w_i(r) \geq d$. Suppose in some round $r' > r$ it is true that $w_i(r') \geq d$. We need to show that $w_i(r'+1) \geq d$. By the definition of *success* applied to round $r'+1$, the number of the ants working on task i at time r' that receive 1 from *success* in round $r'+1$ is $\min\{w_i(r'), d_i\} \geq d$. By the definition of the transition function δ, an ant that receives *success*(1) in round $r'+1$ keeps working on its current task (task i) at time $r'+1$, so $w_i(r'+1) \geq d$. □

Corollary A.2. *For each time r, $|U(r)| \geq |U(r+1)|$ and $|S(r)| \leq |S(r+1)|$.*

For each task $i \in T$ and each time r, let $\Phi_i(r) = \max\{0, (d_i - w_i(r))\}$ be the *deficit* of task i at time r. If $i \in U(r)$, then $\Phi_i(r) = d_i - w_i(r)$. We define the *total deficit* at time r:

$$\Phi(r) = \sum_{i \in T} \Phi_i(r).$$

Lemma A.3. *For each time r and each task $i \in T$, $\Phi_i(r) \geq \Phi_i(r+1)$.*

Proof. Consider fixed task $i \in T$ and time r. If $w_i(r) \geq d_i$, then by Lemma A.1, $w_i(r+1) \geq d_i$, so $\Phi_i(r) = \Phi_i(r+1) = 0$. Otherwise, by Lemma A.1, $w_i(r+1) \geq w_i(r)$, so $\Phi_i(r) = d_i - w_i(r) \geq \max\{0, d_i - w_i(r+1)\} = \Phi_i(r+1)$. In either case, $\Phi_i(r) \geq \Phi_i(r+1)$. □

Corollary A.4. *For each time r, $\Phi(r) \geq \Phi(r+1)$.*

Define an ant to be *inactive* in round r, for $r > 0$, if it is in state $q_\perp$ at time $r-1$ or if it receives *success*(0) in round r. In other words, an ant is inactive if it is not working on any task, or if it unsuccessful at the current task it is working on. So, the number of inactive ants in some round $r+1$ is $|A_\perp(r)| + \sum_{i \in S(r)} (w_i(r) - d_i)$.

Lemma A.5. *The number of inactive ants in round $r+1$ is at least $c \cdot \Phi(r)$.*

Proof. Similarly to the proof of Lemma A.5, the total number of ants $|A|$ can be decomposed into the number of ants not working on any task, the number of ants working on satisfied tasks, and the number of ants working on unsatisfied tasks:

$$|A| = \sum_{i \in U(r)} w_i(r) + \sum_{i \in S(r)} w_i(r) + |A_\perp(r)|.$$

Based on the assumption that $|A| = c \cdot \sum_{i \in T} d_i$, we know that $|A|/c = \sum_{i \in S(r)} d_i + \sum_{i \in U(r)} d_i$. Also, by the definition of $\Phi(r)$, it is true that $\Phi(r) \leq \sum_{i \in T} d_i \leq |A|/c$. The number of inactive ants in round $r+1$ is:

$$\begin{aligned}
|A(r)_\perp| + \sum_{i \in S(r)} (w_i(r) - d_i) &= |A| - \sum_{i \in U(r)} w_i(r) - \sum_{i \in S(r)} w_i(r) + \sum_{i \in S(r)} w_i(r) - \sum_{i \in S(r)} d_i \\
&= |A| - \sum_{i \in U(r)} w_i(r) - \sum_{i \in S(r)} d_i \\
&\geq |A| - \sum_{i \in U(r)} w_i(r) - \left(\frac{|A|}{c} - \sum_{i \in U(r)} d_i \right) \\
&= |A| \left(\frac{c-1}{c} \right) + \sum_{i \in U(r)} (d_i - w_i(r)) \\
&\geq (c-1)\Phi(r) + \Phi(r) = c \cdot \Phi(r).
\end{aligned}$$

□

Next, we show a simple lemma that will be useful in analyzing *choice* components that always provide ants with an unsatisfied task (options (2) and (3)).

Lemma A.6. *Suppose that in each round $r+1$ such that $U(r) \neq \emptyset$, choice returns a task $i \in U(r)$ to each ant. Then, all tasks are satisfied by time T.*

Proof. By Corollary A.2, $|U(r)| \geq |U(r+1)|$ for any r. Therefore, it suffices to show that if $U(r) \neq \emptyset$, then $|U(r)| > |U(r+1)|$. Assume to the contrary that for some time r, such that $|U(r)| \neq 0$, $|U(r)| = |U(r+1)|$. By the definition of an unsatisfied task, $w_i(r+1) < d_i$ for each $i \in U(r)$. By Lemma A.5, the number of inactive ants in round $r+1$ is at least $\Phi(r)$, and, by assumption, *choice* returns an unsatisfied task to each inactive ant. We have the following

contradiction:

$$\begin{aligned}
\Phi(r) &\le \sum_{i \in U(r)} (w_i(r+1) - w_i(r)) && \text{by the specification of } \textit{choice}, \\
&< \sum_{i \in U(r)} (d_i - w_i(r)) && \text{since } w_i(r+1) \le d_i \text{ for each } i \in U(r), \\
&= \Phi(r) && \text{by the definition of } \Phi(r).
\end{aligned}$$

□

The following lemma lets us bound the expected values of the total deficit and the number of unsatisfied tasks given that the probability of satisfying each task is bounded from below.

Lemma A.7. *Suppose that for each unsatisfied task $i \in U(r)$ it is true that $\Pr[w_i(r+1) \ge d_i] \ge p$. Then, $\mathbb{E}[|U(r+1)|] \le |U(r)| \cdot (1-p)$ and $\mathbb{E}[\Phi(r+1)] \le \Phi(r) \cdot (1-p)$.*

Proof. The expected number of unsatisfied tasks at time $r+1$ is:

$$\begin{aligned}
\mathbb{E}\left[|U(r+1)|\right] &\le |U(r)| - \sum_{i \in U(r)} \Pr[w_i(r+1) \ge d_i] \\
&\le |U(r)| - |U(r)| \cdot p \\
&\le |U(r)| \cdot (1-p).
\end{aligned}$$

By assumption, $\Pr[\Phi_i(r+1) \le 0] = \Pr[d_i - w_i(r+1) \le 0] \ge p$. Therefore:

$$\begin{aligned}
\mathbb{E}[\Phi(r+1)] &= \sum_{i \in T} \mathbb{E}[\Phi_i(r+1)] \\
&= \sum_{i \in T} \mathbb{E}[\Phi_i(r+1) \mid \Phi_i(r+1) \le 0] \cdot \Pr[\Phi_i(r+1) \le 0] \\
&\quad + \mathbb{E}[\Phi_i(r+1) \mid \Phi_i(r+1) > 0] \cdot \Pr[\Phi_i(r+1) > 0] \\
&\le \mathbb{E}[\Phi_i(r+1) \mid \Phi_i(r+1) > 0] \cdot \Pr[\Phi_i(r+1) > 0] && \Phi_i(r+1) \ge 0 \\
&\le \Phi_i(r) \cdot (1-p) && \Phi_i(r+1) \le \Phi_i(r).
\end{aligned}$$

□

Next, we analyze the three variations of the *choice* component. In Section B, we analyze the convergence time of task allocation when *choice* returns a uniformly random task, in Section C, we analyze the convergence time of task allocation when *choice* returns a uniformly random *unsatisfied* task, and in Section D, we analyze the convergence time of task allocation when *choice* returns an unsatisfied task with probability proportional to its deficit.

B Uniformly Random Tasks

In this section, we consider the first option for the *choice* component, where in each round *choice* returns a task i with probability $1/|T|$.

For Lemma B.1, B.2 and B.3, assume α is a fixed execution and $r \geq 0$ is some fixed time in α. We consider the state variables at time r and the outputs of *success* in round $r+1$ to be fixed, and we consider the probability distribution over the randomness introduced by the *choice* outputs in round $r+1$.

By Lemma A.5, we know that the number of inactive ants in round $r+1$ is at least $c \cdot \Phi(r)$. By the definition of *choice* in this section, each inactive ant starts working on each task i with probability $1/|T|$. In the next lemma, we show that, in each round, the expected number of new ants to join each unsatisfied task is at least $c \cdot \Phi(r)/|T|$.

Lemma B.1. *For each task $i \in U(r)$, $\mathbb{E}[w_i(r+1) - w_i(r)] \geq c \cdot \Phi(r)/|T|$.*

Proof. Note that, in the expression we want to prove, $w_i(r+1)$ is a random variable, whereas $\Phi(r)$ and $w_i(r)$ are fixed values. By Lemma A.5, the number of inactive ants in round $r+1$ is at least $c \cdot \Phi(r)$. Therefore, for each task i, the expected number of ants that are inactive in round $r+1$ and working on task i at time $r+1$ is:

$$\mathbb{E}[w_i(r+1) - w_i(r)] \geq \frac{c \cdot \Phi(r)}{|T|}.$$

□

After some ants join task i in round $r+1$, it is not guaranteed that the entire new set of ants remains working on task i because some ants may be unsuccessful if task i does not require that many workers. Assuming $c \leq |T|$, since the total deficit is $\Phi(r)$ and there are $|T|$ tasks, we show that the sum of deficits of the top c tasks is at least $c \cdot \Phi(r)/|T|$ (which can be 0 if all tasks are satisfied). Therefore, in expectation, at least $c \cdot \Phi(r)/|T|$ of the new ants that join these tasks will remain working on them. In the next lemma, we show that the expected total deficit $\Phi(r)$ decreases by approximately $c \cdot \Phi(r)/|T|$ in round $r+1$.

Lemma B.2. *For $c \leq |T|$, $\mathbb{E}[\Phi(r+1)] \leq (1 - c/4|T|)\Phi(r)$.*

Proof. The expected decrease in one round of the value of $\Phi(r)$ is:

$$\begin{aligned}
\mathbb{E}[\Phi(r) - \Phi(r+1)] &\geq \sum_{i \in T} \mathbb{E}[\Phi_i(r) - \Phi_i(r+1)] && \text{by Lemma A.3,} \\
&= \sum_{i \in T} \mathbb{E}\left[\min\left\{(w_i(r+1) - w_i(r)), (d_i - w_i(r))\right\}\right] \\
&\geq \frac{1}{4} \sum_{i \in T} \min\left\{\mathbb{E}\left[w_i(r+1) - w_i(r)\right], (d_i - w_i(r))\right\} \\
&\geq \frac{1}{4} \sum_{i \in T} \min\left\{\frac{c \cdot \Phi(r)}{|T|}, (d_i - w_i(r))\right\} && \text{by Lemma B.1.}
\end{aligned}$$

The fourth inequality above is derived by applying Corollary E.2 is to random variable $X = w_i(r+1) - w_i(r)$ and the fixed value $d_i - w_i(r) \geq 1$. Random variable X can be expressed as a sum of k independent binary random variables, where k is the number of inactive ants in round $r+1$ and each ant contributes 1 to the sum if it receives *choice*(i) in round r, and 0 otherwise.

Suppose in contradiction that:

$$\sum_{i \in T} \min \left\{ \frac{c \cdot \Phi(r)}{|T|}, (d_i - w_i(r)) \right\} < \frac{c \cdot \Phi(r)}{|T|}.$$

It must be the case that for each $i \in T$, $d_i - w_i(r) < c \cdot \Phi(r)/|T|$. Since $c \le |T|$, $\sum_{i \in T}(d_i - w_i(r)) < \Phi(r)$, a contradiction.

We have $\mathbb{E}[\Phi(r) - \Phi(r+1)] \ge (1/4)(c \cdot \Phi(r)/|T|)$, so $\mathbb{E}[\Phi(r+1)] \le (1 - c/4|T|)\Phi(r)$. □

Next, we consider the case of $c > |T|$. We can express c as a multiple of $|T|$: $c = c' \cdot |T|$ for some $c' > 1$. Note that c' is not necessarily a constant. We show that in each round, the probability to satisfy each task is at least some constant, and consequently, the expected number of unsatisfied tasks decreases by a constant fraction in each round.

Lemma B.3. *For $c > |T|$, $\mathbb{E}[|U(r+1)|] \le |U(r)|e^{-c'(1-1/c')^2/2}$ and $\mathbb{E}[\Phi(r+1)] \le \Phi(r)e^{-c'(1-1/c')^2/2}$.*

Proof. By Lemma A.5, the number of inactive ants in round $r+1$ is at least $c \cdot \Phi(r)$. Therefore, for each $i \in U(r)$, $\mathbb{E}[w_i(r+1) - w_i(r)] \ge c \cdot \Phi(r)/|T| = c' \cdot \Phi(r)$. By a Chernoff bound it follows that:

$$\begin{aligned}
&\Pr\left[(w_i(r+1) - w_i(r)) < \Phi_i(r)\right] \\
&\quad \le \Pr\left[(w_i(r+1) - w_i(r)) < \Phi(r)\right] \\
&\quad \le \Pr\left[(w_i(r+1) - w_i(r)) < \left(\frac{1}{c'}\right) \mathbb{E}\left[w_i(r+1) - w_i(r)\right]\right] \\
&\quad \le e^{-\frac{\mathbb{E}\left[w_i(r+1) - w_i(r)\right]\left(1 - \frac{1}{c'}\right)^2}{2}} \\
&\quad \le e^{-\frac{c' \cdot \Phi(r)\left(1 - \frac{1}{c'}\right)^2}{2|U(r)|}} \qquad \text{since } \Phi(r) \ge 1, \\
&\quad \le e^{-\frac{c'\left(1 - \frac{1}{c'}\right)^2}{2}}.
\end{aligned}$$

Therefore, $\Pr[w_i(r+1) \ge d_i] \ge 1 - e^{-c'(1-1/c')^2/2}$, so by Lemma A.7, it follows that $\mathbb{E}[|U(r+1)|] \le |U(r)| \cdot e^{-c'(1-1/c')^2/2}$ and $\mathbb{E}[\Phi(r+1)] \le \Phi(r) \cdot e^{-c'(1-1/c')^2/2}$. □

Finally, we fix some arbitrary deterministic *success* components in each round, and we analyze the total running time of task allocation for an arbitrary probabilistic execution of the resulting system. In the next theorem, we start at time 0, when the total deficit is $\Phi(0)$, and inductively apply Lemmas B.2 and B.3 and iterated expectation.

Theorem B.4. *For $c \le |T|$ and for any δ, $0 < \delta < 1$, with probability at least $1 - \delta$, all tasks are satisfied by time $(4/c)|T|(\ln \Phi(0) + \ln(1/\delta))$.*

Proof. First, we show by induction that for each $r \ge 0$ the following holds at time r:

$$\mathbb{E}[\Phi(r)] \le \Phi(0)\left(1 - \frac{c}{4|T|}\right)^r. \tag{1}$$

In the base case, $r = 0$. By Corollary A.4, $\Phi(r) \le \Phi(0)$.

For the inductive step, we assume the statement is true for some fixed $r \ge 0$, and we show it is true for $r+1$. First, consider a fixed prefix α of the execution of length r and some fixed outputs

of the *success* component in round $r+1$. Recall that by assumption, the outputs to all ants of *success* are determined by an arbitrary deterministic component. By Lemma B.2, $\mathbb{E}[\Phi(r+1)] \leq \Phi(r)\,(1 - c/4|T|)$. Note that this expectation has a different meaning from the one in Equation (1) that we want to show. Here, the expectation is taken only over the randomness induced by the *choice* component outputs in round $r+1$ (starting from a fixed time at the end of the execution prefix α), and $\Phi(r)$ is a *fixed value*. Next, we need to state a bound on $\mathbb{E}[\Phi(r+1)]$ over all random choices from the beginning of the probabilistic execution up to time r in terms of the *random variable* $\Phi(r)$. Since all prefixes of length r are disjoint, by the law of total expectation, for all prefixes of length r in the probabilistic execution, it is true that $\mathbb{E}[\Phi(r+1) \mid \Phi(r)] \leq \Phi(r)\,(1 - c/4|T|)$.

Now we use the law of iterated expectation and the inductive hypothesis to obtain a bound on $\mathbb{E}[\Phi(r+1)]$.

$$
\begin{aligned}
\mathbb{E}[\Phi(r+1)] &= \mathbb{E}[\mathbb{E}[\Phi(r+1) \mid \Phi(r)]] && \text{by iterated expectation,}\\
&\leq \mathbb{E}\left[\Phi(r)\left(1 - \frac{c}{4|T|}\right)\right] && \text{by the above bound,}\\
&= \mathbb{E}[\Phi(r)]\left(1 - \frac{c}{4|T|}\right) && \text{by linearity of expectation,}\\
&\leq \Phi(0)\left(1 - \frac{c}{4|T|}\right)^{r+1} && \text{by the inductive hypothesis.}
\end{aligned}
$$

Thus, we have shown that, for every $r \geq 0$, $\mathbb{E}[\Phi(r)] \leq \Phi(0)\,(1 - c/4|T|)^r$.

For $r = (4/c)|T|(\ln \Phi(0) + \ln(1/\delta))$ we get:

$$
\begin{aligned}
\mathbb{E}[\Phi(r)] &\leq \Phi(0)\left(1 - \frac{c}{4|T|}\right)^{r}\\
&\leq \Phi(0) \cdot e^{-\frac{4c|T|(\ln \Phi(0) + \ln(1/\delta))}{4c|T|)}} && \text{by } (1-a)^b \leq e^{-ab} \text{ for } a, b \in \mathbb{R} \text{ and } a, b > 0,\\
&\leq \Phi(0) \cdot e^{-(\ln \Phi(0) + \ln(1/\delta))}\\
&\leq \Phi(0)\left(\frac{1}{\Phi(0)}\right)\delta \leq \delta.
\end{aligned}
$$

By a Markov bound, we get $\Pr[\Phi(r) \geq (1/\delta)\mathbb{E}[\Phi(r)]] \leq \delta$. Therefore, with probability at least $1-\delta$, the total deficit is strictly less than 1 by time $(4/c)|T|(\ln \Phi(0) + \ln(1/\delta))$. This implies that all tasks are satisfied by that time. □

Corollary B.5. *For $c \leq |T|$ and for any δ and ϵ, $0 < \delta, \epsilon < 1$, with probability at least $1-\delta$, the deficit at time $(4/c)|T|(\ln(1/\epsilon) + \ln(1/\delta))$ is at most $\epsilon \cdot \Phi(0)$.*

The proof is similar to the proof of Theorem B.4; we show that $\mathbb{E}[\Phi(r+1)] \leq \Phi(0) \cdot \epsilon \cdot \delta$ instead of $\mathbb{E}[\Phi(r+1)] \leq \delta$.

Theorem B.6. *For $c > |T|$ and for any δ, $0 < \delta < 1$, with probability at least $1-\delta$, all tasks are satisfied by time $(2/(c'(1-1/c')^2))(\min\{\ln|T|, \ln \Phi(0)\} + \ln(1/\delta))$.*

Proof. Initially, $|U(0)| \leq |T|$. By Lemma B.3, $\mathbb{E}[|U(r+1)|] \leq |U(r)| \cdot e^{-\frac{c'\left(1-\frac{1}{c'}\right)^2}{2}}$ and $\mathbb{E}[\Phi(r+1)] \leq \Phi(r) \cdot e^{-\frac{c'\left(1-\frac{1}{c'}\right)^2}{2}}$. Similarly to Theorem B.4, we can inductively apply Lemma B.3 and iterated

expectation to show that for each $r \geq 0$, it is true that $\mathbb{E}[|U(r+1)|] \leq |T| \cdot e^{-rc'(1-1/c')^2/2}$ and $\mathbb{E}[\Phi(r+1)|] \leq \Phi(0) \cdot e^{-rc'(1-1/c')^2/2}$. For $r = (2/(c'(1-1/c')^2))(\min\{\ln|T| + \ln\Phi(0)\} + \ln(1/\delta))$ at least one of the following is true:

$$\mathbb{E}[|U(r)|] \leq |T| \cdot e^{-(2/(c'(1-1/c')^2))(\ln|T|+\ln(1/\delta))} \leq \delta$$
$$\mathbb{E}[\Phi(r)] \leq \Phi(0) \cdot e^{-(2/(c'(1-1/c')^2))(\ln\Phi(0)+\ln(1/\delta))} \leq \delta.$$

Therefore, by a Markov bound, with probability at least $1-\delta$, $|U(r)| < 1$ or $\Phi(r) < 1$, implying that all tasks are satisfied by time $(2/(c'(1-1/c')^2))(\min\{\ln|T|, \ln\Phi(0)\} + \ln(1/\delta))$. □

Corollary B.7. *For $c > |T|$ and for any δ and ϵ, $0 < \delta, \epsilon < 1$, with probability at least $1-\delta$, the deficit at time $(2/(c'(1-1/c')^2))(\ln(1/\epsilon) + \ln(1/\delta))$ is at most $\epsilon \cdot \Phi(0)$.*

The proof is similar to the proof of Theorem B.6; we ignore the case when $|T| < \Phi(0)$ and we show that $\mathbb{E}[\Phi(r+1)] \leq \Phi(0) \cdot \epsilon \cdot \delta$ instead of $\mathbb{E}[\Phi(r+1)] \leq \delta$.

We can combine the results of Theorems B.4 and B.6 by slightly weakening Theorem B.6. Clearly, if c' is extremely close to 1 (that is, c is extremely close to $|T|$), the $1/(c'(1-1/c')^2)$ term in Theorem B.6 becomes very large, and in the limit the running time becomes ∞. Therefore, we can take the result of Theorem B.4 for $c \leq 2|T|$ and the result of Theorem B.6 for $c > 2|T|$, in which case $c' > 2$ and the $2/(c'(1-1/c')^2)$ can be bounded by 4.

Corollary B.8. *For any δ, $0 < \delta < 1$, with probability at least $1-\delta$, all tasks are satisfied by time $\mathcal{O}(c^{-1}) \cdot \mathcal{O}(|T|(\ln\Phi(0) + \ln(1/\delta)))$.*

Similarly, we can combine Corollaries B.5 and B.7.

Corollary B.9. *For any δ and ϵ, $0 < \delta, \epsilon < 1$, with probability at least $1-\delta$, the deficit at time $\mathcal{O}(c^{-1}) \cdot \mathcal{O}(|T|(\ln(1/\epsilon) + \ln(1/\delta)))$ is at most $\epsilon \cdot \Phi(0)$.*

C Uniformly Random Unsatisfied Tasks

Here, we consider the second option for the *choice* component, where in each round *choice* returns a task $i \in U(r)$ with probability $1/|U(r)|$.

The results in this section follow the same structure as the results in Section B. In this section, we do not include as much detail in some of the results. For example, in the proof of Theorem C.5, we skip the detailed application of law of total expectation and iterated expectation since they follow the same pattern as in the proof of Theorem B.4.

For Lemma C.1 and C.2, assume α is a fixed execution and $r \geq 0$ is some fixed time in α. We consider the state variables at time r and the outputs of *success* in round $r+1$ to be fixed, and we consider the probability distribution over the randomness introduced by the *choice* outputs in round $r+1$.

Lemma C.1. *For each unsatisfied task $i \in U(r)$, $\mathbb{E}[w_i(r+1) - w_i(r)] \geq \Phi(r)/|U(r)|$.*

Proof. By Lemma A.5, the number of inactive ants in round $r+1$ is at least $\Phi(r)$. Since the number of unsatisfied tasks is $|U(r)|$, for each unsatisfied task i, it is true that $\mathbb{E}[w_i(r+1) - w_i(r)] \geq \Phi(r)/|U(r)|$. □

We show that in round $r+1$ at least one of the following happens: (1) the total deficit decreases by a constant fraction, or (2) the number of unsatisfied tasks decreases by a constant fraction. To show the first property, we consider tasks with a fairly high deficit, which are not likely to get satisfied in one round. We show that the number of new ants joining such tasks is enough to decrease the total deficit by a constant fraction. To show the second property (the number of unsatisfied tasks decreases by a constant fraction), we focus on tasks with fairly low deficit which are likely to get satisfied within one round. We show that these tasks are enough to decrease the total number of unsatisfied tasks by a constant fraction in one round.

Lemma C.2. *For $c \geq 1$, at least one of the following is true:*

$$\mathbb{E}[\Phi(r+1)] \leq \left(\frac{15}{16}\right)\Phi(r) \tag{2}$$

$$\mathbb{E}[|U(r+1)|] \leq \left(1 - \frac{1-e^{-1/8}}{2}\right)|U(r)| \tag{3}$$

Proof. We define task $i \in U(r)$ to have a *high deficit* if $\Phi_i(r) > \Phi(r)/(2|U(r)|)$. Since the deficit is always an integer, $\Phi_i(r) \geq \lceil \Phi(r)/(2|U(r)|) \rceil$. Otherwise, if $\Phi_i(r) \leq \Phi(r)/(2|U(r)|)$ for a task $i \in U(r)$, task i is defined to have a *low deficit.* Let $H(r)$ be the set of high-deficit tasks and let $L(r) = U(r) \setminus H(r)$ be the set of low-deficit tasks. We consider two cases based on the value of $|H(r)|$.

Case 1: $|H(r)| \geq |U(r)|/2$. The expected decrease of $\Phi(r)$ after one round is:

$$\begin{aligned}
\mathbb{E}[\Phi(r) - \Phi(r+1)] &\geq \sum_{i \in H(r)} \mathbb{E}[\Phi_i(r) - \Phi_i(r+1)] && \text{by Lemma A.3,} \\
&= \sum_{i \in H(r)} \mathbb{E}\left[\min\left\{(w_i(r+1) - w_i(r)), (d_i - w_i(r))\right\}\right] \\
&\geq \sum_{i \in H(r)} \mathbb{E}\left[\min\left\{(w_i(r+1) - w_i(r)), \left\lceil \frac{\Phi(r)}{2|U(r)|} \right\rceil\right\}\right] \\
&\geq \sum_{j \in H(r)} \frac{1}{4} \min\left\{\mathbb{E}\left[w_i(r+1) - w_i(r)\right], \left\lceil \frac{\Phi(r)}{2|U(r)|} \right\rceil\right\} \\
&\geq \sum_{j \in H(r)} \frac{1}{4} \min\left\{\frac{\Phi(r)}{|U(r)|}, \left\lceil \frac{\Phi(r)}{2|U(r)|} \right\rceil\right\} && \text{by Lemma C.1,} \\
&= \frac{|H(r)|}{4} \cdot \frac{\Phi(r)}{2|U(r)|} \\
&\geq \frac{|U(r)|}{8} \cdot \frac{\Phi(r)}{2|U(r)|} \geq \frac{\Phi(r)}{16}.
\end{aligned}$$

In this case, Equation (2) is satisfied.

Case 2: $|H(r)| < |U(r)|/2$, so $|L(r)| \geq |U(r)|/2$. In this case, many of the tasks have low deficit and each one of them is fairly close to being satisfied.

Consider a low-deficit task $i \in L(r)$. By Lemma C.1, $\mathbb{E}\left[w_i(r+1) - w_i(r)\right] \geq \Phi(r)/|U(r)|$. Also,

by definition, $\Phi(r) \geq |U(r)|$, so $\mathbb{E}[w_i(r+1) - w_i(r)] \geq 1$. Applying a Chernoff bound, we get:

$$
\begin{aligned}
&\Pr\left[(w_i(r+1) - w_i(r)) < \frac{\Phi(r)}{2|U(r)|}\right] \\
&\leq \Pr\left[(w_i(r+1) - w_i(r)) < \left(\frac{1}{2}\right)\mathbb{E}[w_i(r+1) - w_i(r)]\right] \\
&\leq e^{-\mathbb{E}[w_i(r+1)-w_i(r)]/8} && \text{since } \mathbb{E}[w_i(r+1) - w_i(r)] \geq 1, \\
&\leq e^{-1/8}.
\end{aligned}
$$

Thus, the probability task i is satisfied at time $r+1$ is:

$$
\begin{aligned}
\Pr[w_i(r+1) \geq d_i] &= \Pr[(w_i(r+1) - w_i(r)) \geq (d_i - w_i(r))] \\
&\geq \Pr\left[(w_i(r+1) - w_i(r)) \geq \frac{\Phi(r)}{2|U(r)|}\right] && \text{since } i \in L(r), \\
&\geq 1 - e^{-1/8} && \text{by the inequality above.}
\end{aligned}
$$

The number of newly satisfied tasks in round r is at least $\sum_{i \in L(r)} \Pr[w_i(r+1) \geq d_i]$ in expectation. Therefore, the expected number of unsatisfied tasks at time $r+1$ is:

$$
\begin{aligned}
\mathbb{E}[|U(r+1)|] &\leq |U(r)| - \sum_{i \in L(r)} \Pr[w_i(r+1) \geq d_i] \\
&\leq |U(r)| - \frac{|U(r)|}{2}\left(1 - e^{-1/8}\right) \\
&\leq |U(r)|\left(1 - \frac{1 - e^{-1/8}}{2}\right).
\end{aligned}
$$

In this case, Equation (3) holds. □

Next, we consider the case of $c > 1$. Let d and k be arbitrary constants such that $0 < d < 1 - 1/c$ (so $c(1-d) > 1$) and $k = (1 - 1/c(1-d))(1 - e^{-cd^2/2})$.

Lemma C.3. *For $c > 1$ and for each unsatisfied task $i \in U(r)$, $\Pr[(w_i(r+1) - w_i(r)) < (1-d)c \cdot \Phi(r)/|U(r)|] \leq e^{-cd^2/2}$.*

Proof. By Lemma A.5, the number of inactive ants in round $r+1$ is at least $c \cdot \Phi(r)$. Therefore, for each $i \in U(r)$, $\mathbb{E}[w_i(r+1) - w_i(r)] \geq c \cdot \Phi(r)/|U(r)|$. By a Chernoff bound it follows that:

$$
\begin{aligned}
&\Pr\left[(w_i(r+1) - w_i(r)) < \frac{(1-d)c\Phi(r)}{|U(r)|}\right] \\
&\leq \Pr[(w_i(r+1) - w_i(r)) < (1-d)\mathbb{E}[w_i(r+1) - w_i(r)]] \\
&\leq e^{-\frac{\mathbb{E}[w_i(r+1)-w_i(r)]d^2}{2}} \\
&\leq e^{-\frac{c \cdot \Phi(r) d^2}{2|U(r)|}} && \text{since } \Phi(r)/|U(r)| \geq 1, \\
&\leq e^{-\frac{cd^2}{2}}.
\end{aligned}
$$

□

Unlike Theorem C.5, where in round $r+1$ either the total deficit or the number of unsatisfied tasks decreases by a constant fraction, here we show that the number of unsatisfied tasks decreases by at least a constant fraction in round $r+1$ (this roughly corresponds to Case 2 in Theorem C.5). We consider all tasks with a fairly low deficit, which are likely to get satisfied in a single round. The total deficit at time r is $\Phi(r)$, and the total number of inactive ants in round $r+1$ is at least $c \cdot \Phi(r)$. The fact that the number of inactive ants is at least a constant fraction greater than total deficit lets us show that the expected number of low-deficit tasks is at least a constant fraction of all unsatisfied tasks. Therefore, by satisfying these low-deficit tasks the number of unsatisfied tasks decreases by a constant fraction in expectation.

Lemma C.4. *For $c > 1$, $\mathbb{E}[|U(r+1)|] \leq |U(r)|(1-k)$.*

Proof. Define task $i \in U(r)$ to have a *low deficit* if $\Phi_i(r) \leq (1-d)c\Phi(r)/|U(r)|$, and let $L(r) \subseteq U(r)$ denote the set of low-deficit tasks at time r. Similarly, let task $i \in U(r)$ have a *high deficit* if $\Phi_i(r) > (1-d)c\Phi(r)/|U(r)|$, and let $H(r) \subseteq U(r)$ denote the set of high-deficit tasks at time r. Therefore, $|U(r)| = |L(r)| + |H(r)|$.

Since the total deficit at time r is $\Phi(r)$, and each high-deficit task has deficit at least $(1-d)c\Phi(r)/|U(r)|$, it must be the case that:

$$|H(r)| \leq \frac{\Phi(r)}{(1-d)c\Phi(r)/|U(r)|} = \frac{|U(r)|}{c(1-d)}.$$

Therefore,

$$|L(r)| = |U(r)| - |H(r)| \geq |U(r)| \left(1 - \frac{1}{c(1-d)}\right).$$

For a task i with low deficit, the probability that it is satisfied at time $r+1$ is:

$$\begin{aligned}
\Pr\left[w_i(r+1) \geq d_i\right] &= \Pr\left[(w_i(r+1) - w_i(r)) \geq (d_i - w_i(r))\right] \\
&\geq \Pr\left[(w_i(r+1) - w_i(r)) \geq \frac{(1-d)c\Phi(r)}{|U(r)|}\right] \\
&\geq 1 - e^{-\frac{cd^2}{2}} \qquad \text{by Lemma C.3.}
\end{aligned}$$

Therefore, the expected number of unsatisfied tasks at time $r+1$ is:

$$\begin{aligned}
\mathbb{E}\left[|U(r+1)|\right] &= |U(r)| - \sum_{i \in L(r)} \Pr\left[w_i(r+1) \geq d_i\right] \\
&\leq |U(r)| - |U(r)| \left(1 - \frac{1}{c(1-d)}\right)\left(1 - e^{-\frac{cd^2}{2}}\right) \\
&= |U(r)| \left(1 - \left(1 - \frac{1}{c(1-d)}\right)\left(1 - e^{-\frac{cd^2}{2}}\right)\right) \\
&= |U(r)|\left(1-k\right).
\end{aligned}$$

□

Finally, we analyze the total running time of task allocation for an arbitrary probabilistic execution of the resulting system. Fix some arbitrary deterministic *success* components in each round. In the next theorem, we start at time 0, when the total deficit is $\Phi(0)$ and the number of unsatisfied tasks is at most $|T|$, and inductively apply Lemmas C.2 and C.4 and iterated expectation.

Theorem C.5. *For $c \geq 1$ and for any δ, $0 < \delta < 1$, with probability at least $1 - \delta$, all tasks are satisfied by time* $\min\{|T|, 32(\ln \Phi(0) + \ln |T| + \ln(1/\delta))\}$.

Proof. By Corollary A.2 and A.4, both the number of unsatisfied tasks and the total deficit are monotonically non-increasing. Initially, the total deficit is $\Phi(0)$ and $|U(0)| \leq |T|$. Informally, by Lemma C.2, in each round, either the number of unsatisfied tasks or the total deficit decreases by a constant fraction. So, if we consider r rounds, then either in at least $r/2$ rounds the total deficit decreases by a constant fraction, or in at least $r/2$ rounds the number of unsatisfied tasks decreases by a constant fraction. Formally, similarly to Theorem B.4, we can inductively apply either Equation 2 or Equation 3 together with iterated expectation to show that for each $r \geq 0$, at least one of the following is true:

$$\mathbb{E}[|U(r)|] \leq |T| \left(1 - \frac{1 - e^{-1/8}}{2}\right)^{r/2}$$

$$\mathbb{E}[|\Phi(r)|] \leq \Phi(0) \left(1 - \frac{1}{16}\right)^{r/2}.$$

Therefore, for $r = 32(\ln \Phi(0) + \ln |T| + \ln(1/\delta))$, at least one of the following is true:

$$\mathbb{E}[|U(r)|] \leq |T| \left(1 - \frac{1 - e^{-1/8}}{2}\right)^{16(\ln \Phi(0) + \ln |T| + \ln(1/\delta))} \leq \delta$$

$$\mathbb{E}[|\Phi(r)|] \leq \Phi(0) \left(1 - \frac{1}{16}\right)^{16(\ln \Phi(0) + \ln |T| + \ln(1/\delta))} \leq \delta.$$

By a Markov bound, with probability at least $1 - \delta$, either $|U(r)| < 1$ or $\Phi(r) < 1$, implying that all tasks are satisfied by time r. Since *choice* always returns an unsatisfied task, by Lemma A.6, all tasks are satisfied by time $|T|$. So, overall, with probability at least $1 - \delta$, all tasks are satisfied by time $\min\{|T|, 32(\ln \Phi(0) + \ln |T| + \ln(1/\delta))\}$. □

Corollary C.6. *For $c \geq 1$ and for any δ and ϵ, $0 < \delta, \epsilon < 1$, with probability at least $1 - \delta$, the deficit at time* $\min\{|T|, 32(\ln |T| + \ln(1/\epsilon) + \ln(1/\delta))\}$ *is at most* $\epsilon \cdot \Phi(0)$.

Theorem C.7. *For $c > 1$ and for any δ, $0 < \delta < 1$, with probability at least $1 - \delta$, all tasks are satisfied by time* $\min\{|T|, (2/\ln c)(\ln |T| + \ln(1/\delta))\}$.

Proof. Initially, $|U(0)| \leq |T|$. By Lemma C.4, $\mathbb{E}[|U(r + 1)|] \leq |U(r)|(1 - k)$. Similarly to Theorem B.4, we can inductively apply Lemma C.4 and iterated expectation to show that for each $r \geq 0$, it is true that $\mathbb{E}[|U(r + 1)|] \leq |T|(1 - k)^r$. For $r = (\ln(1 - k)^{-1})^{-1}(\ln |T| + \ln(1/\delta))$ we have:

$$\mathbb{E}[|U(r)|] \leq |T|(1 - k)^{(\ln(1-k)^{-1})^{-1}(\ln |T| + \ln(1/\delta))} \leq \delta.$$

Therefore, by a Markov bound, with probability at least $1 - \delta$, $|U(r)| < 1$, implying that all tasks are satisfied by time $(\ln(1 - k)^{-1})^{-1}(\ln |T| + \ln(1/\delta))$. Since *choice* always returns an unsatisfied task, by Lemma A.6, all tasks are satisfied by time $|T|$, and the lemma follows. □

We can combine the results of Theorems C.5 and C.7. Clearly, if c is extremely close to 1, the $2/\ln c$ term becomes very large, and in the limit the running time becomes ∞. Therefore, we can take the minimum of the running times of Theorems C.5 and C.7 to get the overall running time of the algorithm.

Corollary C.8. *For any δ, $0 < \delta < 1$, with probability at least $1 - \delta$, all tasks are satisfied by time* $\min\{|T|, \mathcal{O}(\ln^{-1} c) \cdot \mathcal{O}(\min\{|T|, \ln \Phi(0) + \ln |T| + \ln(1/\delta))\}\}$.

Corollary C.9. *For any δ and ϵ, $0 < \delta, \epsilon < 1$, with probability at least $1 - \delta$, the deficit at time* $\min\{|T|, \mathcal{O}(\ln^{-1} c) \cdot \mathcal{O}(\ln(1/\epsilon) + \ln |T| + \ln(1/\delta))\}$ *is at most $\epsilon \cdot \Phi(0)$.*

D Unsatisfied Tasks Prioritized by Deficit

In this section, we consider the third option for the *choice* component, where in each round *choice* returns a task $i \in U(r)$ with probability $(d_i - w_i(r))/\Phi(r)$. In Section D.1, we analyze the time for ants to re-allocate when alternative tasks in each round are determined based on option (3). Then, in Section D.2, we present an alternative model where each of the *success* and *choice* components provides noisy information to the ants. For the resulting noisy variant of option (3), we analyze the time for ants to re-allocate, satisfying the demands of the tasks approximately.

D.1 Option (3) with no Uncertainty

For Lemmas D.1 and D.2, assume α is a fixed execution and $r \geq 0$ is some fixed time in α. We consider the state variables at time r and the outputs of *success* in round $r+1$ to be fixed, and we consider the probability distribution over the randomness introduced by the *choice* outputs in round $r+1$.

Since an inactive ant starts working on a task i with probability $(d_i - w_i(r))/\Phi(r)$, and since there are at least $\Phi(r)$ inactive ants in round $r+1$, the expected number of new ants to join task i in round $r+1$ is at least a constant fraction of $d_i - w_i(r)$, which is exactly the deficit of the task at time r. In the next lemma, we show that each task is satisfied in round $r+1$ with a constant probability, and so the total number of unsatisfied tasks decreases by at least a constant fraction.

Lemma D.1. *For $c \geq 1$, $\mathbb{E}[|U(r+1)|] \leq |U(r)|/2$ and $\mathbb{E}[\Phi(r+1)] \leq \Phi(r)/2$.*

Proof. We start by bounding the probability $\Pr[w_i(r+1) \geq d_i]$ from below, for some task $i \in U(r)$. We can express $(w_i(r+1) - w_i(r))$ as a binomial variable that is the sum of n independent identical random variables, each with probability p of success. By Lemma A.5, the number of inactive ants in round $r+1$ is at least $\Phi(r)$, so $n \geq \Phi(r)$, and by the definition of *choice*, we know that $p = (d_i - w_i(r))/\Phi(r)$. By [1], the median m of $(w_i(r+1) - w_i(r))$ is:

$$\left\lfloor \frac{n(d_i - w_i(r))}{\Phi(r)} \right\rfloor \leq m \leq \left\lceil \frac{n(d_i - w_i(r))}{\Phi(r)} \right\rceil .$$

Since we want to lower-bound $\Pr[w_i(r+1) - w_i(r) \geq d_i - w_i(r)]$, we can consider $n = \Phi(r)$ because the probability that task i is satisfied only increases if we increase n. Therefore, $m = d_i - w_i(r)$, and by the definition of the median it follows that $\Pr[w_i(r+1) - w_i(r) \geq d_i - w_i(r)] \geq 1/2$.

By Lemma A.7, $\mathbb{E}[|U(r+1)|] \leq |U(r)|/2$ and $\mathbb{E}[\Phi(r+1)] \leq \Phi(r)/2$. □

Next, we consider the case of $c > 1$. Similarly to Section C, we show that each task is satisfied with a constant probability, so the number of unsatisfied tasks and the total deficit decrease by a constant fraction in each round.

Lemma D.2. *For $c > 1$, $\mathbb{E}[|U(r+1)|] \leq |U(r)| \cdot e^{-c(1-1/c)^2/2}$ and $\mathbb{E}[\Phi(r+1)] \leq \Phi(r) \cdot e^{-c(1-1/c)^2/2}$.*

Proof. By Lemma A.5, the number of inactive ants in round $r+1$ is at least $c \cdot \Phi(r)$. Therefore, for each $i \in U(r)$, $\mathbb{E}[w_i(r+1) - w_i(r)] \geq c \cdot \Phi(r)(d_i - w_i(r))/\Phi(r) = c \cdot (d_i - w_i(r))$. By a Chernoff bound it follows that:

$$
\begin{aligned}
&\Pr\left[(w_i(r+1) - w_i(r)) < (d_i - w_i(r))\right] \\
&\leq \Pr\left[(w_i(r+1) - w_i(r)) < \left(\frac{1}{c}\right)\mathbb{E}\left[w_i(r+1) - w_i(r)\right]\right] \\
&\leq e^{-\frac{\mathbb{E}[w_i(r+1)-w_i(r)]\left(1-\frac{1}{c}\right)^2}{2}} \\
&\leq e^{-\frac{c\cdot(d_i - w_i(r))\left(1-\frac{1}{c}\right)^2}{2|U(r)|}} \qquad \text{since } (d_i - w_i(r)) \geq 1, \\
&\leq e^{-\frac{c\left(1-\frac{1}{c}\right)^2}{2}}.
\end{aligned}
$$

By Lemma A.7, $\mathbb{E}[|U(r+1)|] \leq |U(r)| \cdot e^{-c(1-1/c)^2/2}$ and $\mathbb{E}[\Phi(r+1)] \leq \Phi(r) \cdot e^{-c(1-1/c)^2/2}$. □

Finally, we fix some arbitrary deterministic *success* components in each round and we analyze the total running time of task allocation for an arbitrary probabilistic execution of the resulting system. In the next theorem, we start at time 0, when the number of unsatisfied tasks is at most $|T|$, and inductively apply Lemmas D.1 and D.2 and iterated expectation.

Theorem D.3. *For $c \geq 1$ and for any δ, $0 < \delta < 1$, with probability at least $1 - \delta$, all tasks are satisfied by time* $\min\{|T|, \min\{\log|T|, \log\Phi(0)\} + \log(1/\delta)\}$.

Proof. Initially, $|U(0)| \leq |T|$. By Lemma D.1, $\mathbb{E}[|U(r+1)|] \leq |U(r)|/2$ and $\mathbb{E}[\Phi(r+1)] \leq \Phi(r)/2$. Similarly to Theorem B.4, we can inductively apply Lemma D.1 and iterated expectation to show that for each $r \geq 0$, it is true that $\mathbb{E}[|U(r+1)|] \leq |T|(1/2)^r$ and $\mathbb{E}[\Phi(r+1)] \leq \Phi(0)(1/2)^r$. For $r = \min\{\log|T| + \log\Phi(0)\} + \log(1/\delta)$ we have that at least one of the following is true:

$$
\begin{aligned}
\mathbb{E}[|U(r)|] &\leq |T| \cdot 2^{-(\min\{\log|T|, \Phi(0)\} + \log(1/\delta))} \leq \delta \\
\mathbb{E}[\Phi(r)] &\leq \Phi(0) \cdot 2^{-(\min\{\log|T|, \Phi(0)\} + \log(1/\delta))} \leq \delta.
\end{aligned}
$$

Therefore, by a Markov bound, with probability at least $1 - \delta$, either $|U(r)| < 1$ or $\Phi(0) < 1$, implying that all tasks are satisfied by time $\min\{\log|T|, \log\Phi(0)\} + \log(1/\delta)$. Since *choice* always returns an unsatisfied task, by Lemma A.6, all tasks are satisfied by time $|T|$, and the lemma follows. □

Corollary D.4. *For $c \geq 1$ and for any δ and ϵ, $0 < \delta, \epsilon < 1$, with probability at least $1 - \delta$, the deficit at time* $\min\{|T|, \log(1/\epsilon) + \log(1/\delta)\}$ *is at most* $\epsilon \cdot \Phi(0)$.

Theorem D.5. *For $c > 1$ and for any δ, $0 < \delta < 1$, with probability at least $1 - \delta$, all tasks are satisfied by time* $\min\{|T|, (2/(c(1-1/c)^2))(\min\{\ln|T|, \ln\Phi(0)\} + \ln(1/\delta))\}$.

Proof. Initially, $|U(0)| \leq |T|$. By Lemma D.2, $\mathbb{E}[|U(r+1)|] \leq |U(r)| \cdot e^{-\frac{c\left(1-\frac{1}{c}\right)^2}{2}}$ and $\mathbb{E}[\Phi(r+1)] \leq \Phi(r) \cdot e^{-\frac{c\left(1-\frac{1}{c}\right)^2}{2}}$. Similarly to Theorem B.4, we can inductively apply Lemma D.1 and iterated expectation to show that for each $r \geq 0$, it is true that $\mathbb{E}[|U(r+1)|] \leq |T|(e^{-rc(1-1/c)^2/2})$ and

$\mathbb{E}[\Phi(r+1)] \leq \Phi(0)(e^{-rc(1-1/c)^2/2})$. For $r = (2/(c(1-1/c)^2))(\min\{\ln|T|, \ln\Phi(0)\} + \ln(1/\delta))$ we have that at leas one of the following is true:

$$\mathbb{E}[|U(r)|] \leq |T| \cdot e^{-(2/(c(1-1/c)^2))(\min\{\ln|T|,\ln\Phi(0)\}+\ln(1/\delta))} \leq \delta$$
$$\mathbb{E}[\Phi(r)] \leq \Phi(0) \cdot e^{-(2/(c(1-1/c)^2))(\min\{\ln|T|,\ln\Phi(0)\}+\ln(1/\delta))} \leq \delta.$$

Therefore, by a Markov bound, with probability at least $1-\delta$, either $|U(r)| < 1$ or $\Phi(r) < 1$, implying that by time $(2/(c(1-1/c)^2))(\min\{\ln|T|, \ln\Phi(0)\} + \ln(1/\delta))$ all tasks are satisfied. Since *choice* always returns an unsatisfied task, by Lemma A.6, all tasks are satisfied by time $|T|$, and the lemma follows. □

Corollary D.6. *For $c > 1$ and for any δ and ϵ, $0 < \delta, \epsilon < 1$, with probability at least $1-\delta$, the deficit at time $\min\{|T|, (2/(c(1-1/c)^2))(\log(1/\epsilon) + \log(1/\delta))\}$ is at most $\epsilon \cdot \Phi(0)$.*

We can combine the results of Theorems D.3 and D.5. Clearly, if c is extremely close to 1, the $1/(c(1-1/c)^2)$ term becomes very large, and in the limit the running time becomes ∞. Therefore, we can take the minimum of the running times of Theorems D.3 and D.5 to get the overall running time of the algorithm.

Corollary D.7. *For any δ, $0 < \delta < 1$, with probability at least $1-\delta$, all tasks are satisfied by time $\min\{|T|, \mathcal{O}(c^{-1}) \cdot \mathcal{O}(\log\Phi(0) + \log(1/\delta))\}$.*

For $c = 2 + \sqrt{3} \approx 3.7$, we have $2/(c(1-1/c)^2) = 1$, so we can think of the running time being bounded by Theorem D.3 for $c \in [1, 2+\sqrt{3}]$ and bounded by Theorem D.5 for $c > 2+\sqrt{3}$.

Corollary D.8. *For any δ and ϵ, $0 < \delta, \epsilon < 1$, with probability at least $1-\delta$, the deficit at time $\min\{|T|, \mathcal{O}(c^{-1}) \cdot \mathcal{O}(\log(1/\epsilon) + \log(1/\delta))\}$ is at most $\epsilon \cdot \Phi(0)$.*

D.2 Option (3) under Uncertainty

Suppose the *success* component is not completely reliable and it can flip the 0/1 bits of at most $0 \leq z \leq |A|$ ants in round $r+1$. Moreover, we assume the information needed to determine the outputs of the *choice* component in the same round is based on the state variables at time r. That is, the outputs of *choice* in round $r+1$ do not incorporate the outputs of *success* (with the z potential mistakes) in round $r+1$.

Also, suppose the *choice* component is also not completely reliable and can change the probability of outputting task i from exactly $\Phi_i(r)/\Phi(r)$ to any value larger than $(1-y)(\Phi_i(r)/\Phi(r))$ for any $0 \leq y < 1$ while still maintaining a probability distribution over all the tasks.

In other words, we have an alternative model where we consider two types of uncertainty: the number of successful ants and the probabilities with which tasks are assigned to ants are not exact but bounded.

Next, we analyze the time for ants to re-allocate in this modified model. The statements and proofs below are very similar to the ones in Section D.1 with the following two main differences. First, it is no longer possible to guarantee that all tasks are satisfied but we can show that the deficit does not exceed z. Second, whenever the *choice* component is supposed to return a given task with some probability p, we use the lower bound $p(1-y)$ for that probability; this results in a running time that increases as y approaches 1.

For Lemmas D.9 and D.10, assume α is a fixed execution and $r \geq 0$ is some fixed time in α. We consider the state variables at time r and the outputs of *success* in round $r+1$ to be fixed, and we consider the probability distribution over the randomness introduced by the *choice* outputs in round $r+1$.

For each task $i \in T$, let z_i^0 be the number of 0's flipped to 1's, and let z_i^1 be the number of 1's flipped to 0's by *success* in round $r+1$. Let $z^0 = \sum_{i \in T} z_i^0$ and $z^1 = \sum_{i \in T} z_i^1$, so $z^0 + z^1 \leq z$.

Note that, based on the definitions above, the number of workers $w_i(r)$ working at task i decreases by z_i^1 before the unsuccessful ants choose a new task to join. Also, the number of inactive ants is at least $\Phi(r) - z_i^0$ because z_i^0 ants are informed they are successful while they are actually not successful.

Lemma D.9. *For $c \geq 1$, $\mathbb{E}[\Phi(r+1)] \leq (1/4)((3+y)\Phi(r) + z)$.*

Proof. Let random variable $X_i(r+1)$ denote the number of ants that join task i in round $r+1$. The probability for an ants to receive task i from *choice* in round $r+1$ is $p_i \in [(1-y)(\Phi_i(r)/\Phi(r)), (1+y)(\Phi_i(r)/\Phi(r))]$. By Lemma A.5, the number of inactive ants in round $r+1$ is at least $\Phi(r)$; however, now they may be fewer if *success* flipped some 0's to 1's, so the number of inactive ants is at least $\Phi(r) - z^0$. So, $\mathbb{E}[X_i(r+1)] \geq p_i \cdot (\Phi(r) - z^0)$ and the expected value of $\Phi(r+1)$ is:

$$\begin{aligned}
\mathbb{E}[\Phi(r) - \Phi(r+1)] &= \sum_{i \in T} \mathbb{E}[\min\{X_i(r+1) - z_i^1, (d_i - w_i(r))\}] \\
&\geq \sum_{i \in T} \left(\frac{1}{4}\right) \min\{\mathbb{E}[X_i(r+1)] - z_i^1, (d_i - w_i(r))\} \\
&\geq \sum_{i \in T} \left(\frac{1}{4}\right) \min\left\{p_i \cdot (\Phi(r) - z^0) - z_i^1, \Phi_i(r)\right\} \\
&\geq \left(\frac{1}{4}\right) \sum_{i \in T} (1-y) \cdot \Phi_i(r) - p_i \cdot z^0 - z_i^1 \\
&\geq \left(\frac{1}{4}\right) \left((1-y) \cdot \Phi(r) - z^0 - z^1\right) \\
&\geq \left(\frac{1}{4}\right) \left((1-y) \cdot \Phi(r) - z\right).
\end{aligned}$$

□

Lemma D.10. *For $c > 1$, $\mathbb{E}[\Phi(r) - \Phi(r+1)] \geq (1 - e^{-(c(1-1/c)^2)/2})((1-y) \cdot \Phi(r) - z)$.*

Proof. Let that random variable $X_i(r+1)$ denote the number of ants that join task i in round $r+1$. We assumed that the outputs of *choice* are based only on $w_i(r)$, so the probability for an ants to receive task i from *choice* in round $r+1$ is $p_i \in [(1-y)(\Phi_i(r)/\Phi(r)), (1+y)(\Phi_i(r)/\Phi(r))]$ regardless of the outputs of *success* in round $r+1$. By Lemma A.5, the number of inactive ants in round $r+1$ is at least $c \cdot \Phi(r)$. However, after the mistakes of the *success* component, the number of inactive ants is at least $c \cdot \Phi(r) - z^0 \geq c \cdot (\Phi(r) - z^0)$. So, $\mathbb{E}[X_i(r+1)] \geq p_i c \cdot (\Phi(r) - z^0)$.

By a Chernoff bound, it follows that:

$$\Pr\left[X_i(r+1) < p_i(\Phi(r) - z^0)\right] \leq \Pr\left[X_i(r+1) < \left(\frac{1}{c}\right) \mathbb{E}[X_i(r+1)]\right] < e^{-\frac{c(1-\frac{1}{c})^2}{2}}.$$

By linearity of expectation it follows that:

$$\begin{aligned}
\mathbb{E}[\Phi(r) - \Phi(r+1)] &= \sum_{i \in T} \mathbb{E}[\Phi_i(r) - \Phi_i(r+1)] \\
&\geq \sum_{i \in T} \mathbb{E}[\Phi_i(r) - \Phi_i(r+1) \mid X_i(r+1) > p_i(\Phi(r) - z^0)] \\
&\qquad\qquad\qquad \cdot \Pr[X_i(r+1) > p_i(\Phi(r) - z^0)] \\
&\geq \sum_{i \in T} \min\{p_i(\Phi(r) - z^0) - z_i^1, \Phi_i(r)\} \\
&\qquad\qquad\qquad \cdot \Pr[X_i(r+1) > p_i(\Phi(r) - z^0)] \\
&\geq \sum_{i \in T} \left(p_i(\Phi(r) - z^0) - z_i^1\right) \cdot \left(1 - e^{-\frac{c(1-\frac{1}{c})^2}{2}}\right) \\
&\geq \left(1 - e^{-\frac{c(1-\frac{1}{c})^2}{2}}\right) \sum_{i \in T} (1-y)\Phi_i(r) - p_i z^0 - z_i^1 \\
&\geq \left(1 - e^{-\frac{c(1-\frac{1}{c})^2}{2}}\right) ((1-y)\Phi(r) - z).
\end{aligned}$$

□

Finally, we fix some arbitrary deterministic *success* components in each round and we analyze the total running time of task allocation for an arbitrary probabilistic execution of the resulting system. In the next theorem, we start at time 0 and inductively apply Lemmas D.9 and D.10 and iterated expectation.

Theorem D.11. *For $c \geq 1$, for any δ, $0 < \delta < 1$, and for $r = (1/\ln(4/(3+y)))(\ln \Phi(0) + \ln(1/\delta))$, $\Pr[\Phi(r) \leq z] \geq 1 - \delta$.*

Proof. By Lemma D.9, $\mathbb{E}[\Phi(r+1)] \leq (1/4)((3+y)\Phi(r) + z)$. Similarly to Theorem B.4, we can inductively apply Lemma D.9 and iterated expectation to show that for each $r \geq 0$, it is true that:

$$\mathbb{E}[\Phi(r+1)] \leq \Phi(0) \left(\frac{3+y}{4}\right)^r + z \left(\frac{(4/(1-y))^r - 1}{(4/(1-y))^r}\right) \leq \Phi(0) \left(\frac{3+y}{4}\right)^r + z.$$

For $r = (1/\ln(4/(3+y)))(\ln \Phi(0) + \ln(1/\delta))$ we have that:

$$\mathbb{E}[\Phi(r) - z] \leq \Phi(0) \cdot \left(\frac{3+y}{4}\right)^{(1/\ln(4/(3+y)))(\ln \Phi(0) + \ln(1/\delta))} \leq \delta.$$

Therefore, by a Markov bound, $\Pr[\Phi(r) - z \geq 1] \leq \delta$, so $\Pr[\Phi(r) - z < 1] \geq 1 - \delta$, implying that with probability at least $1 - \delta$, $\Phi(r) - z \leq 0$, and so $\Pr[\Phi(r) \leq z] \geq 1 - \delta$. □

Let $k = (1-y)(1 - e^{-(c(1-1/c)^2)/2})$.

Theorem D.12. *For $c > 1$, for any δ, $0 < \delta < 1$, and for round $r = (\ln(1-k)^{-1})^{-1}(\ln \Phi(0) + \ln(1/\delta))$, $\Pr[\Phi(r) \leq z] \geq 1 - \delta$.*

Proof. Similarly to Theorem D.11, we can inductively apply Lemma D.10 and iterated expectation to show that for each $r \geq 0$, it is true that:

$$\mathbb{E}[\Phi(r+1)] \leq \Phi(0)\left(1-(1-y)\left(1-e^{-\frac{c(1-\frac{1}{c})^2}{2}}\right)\right)^r + z = \Phi(0)\cdot(1-k)^r + z.$$

For $r = (\ln(1-k)^{-1})^{-1}(\ln\Phi(0) + \ln(1/\delta))$ we have that:

$$\mathbb{E}[\Phi(r) - z] \leq \Phi(0)\cdot(1-k)^r \leq \Phi(0)\cdot e^{-(\ln\Phi(0)+\ln(1/\delta))} \leq \delta.$$

Therefore, by a Markov bound, $\Pr[\Phi(r) - z \geq 1] \leq \delta$, so $\Pr[\Phi(r) - z < 1] \geq 1-\delta$, implying that with probability at least $1-\delta$, $\Phi(r) - z \leq 0$, and so $\Pr[\Phi(r) \leq z] \geq 1-\delta$. □

Corollary D.13. *For any δ, $0 < \delta < 1$, and for $r = \min\{1/\ln(4/(3+y)), (\ln(1-k)^{-1})^{-1}\}(\ln\Phi(0) + \ln(1/\delta)) = \mathcal{O}(\max\{c^{-1}, \ln^{-1}(y^{-1})\})(\ln\Phi(0) + \ln(1/\delta))$, $\Pr[\Phi(r) \leq z] \geq 1-\delta$.*

E Math Preliminaries

Lemma E.1. *For each $k \geq 1$, let $I_1, \cdots, I_k$ be identically distributed independent binary random variables, and let $X = \sum_{i=1}^{k} I_i$. For an arbitrary constant $c > 0$:*

$$\mathbb{E}[\min\{X, c\}] \geq \frac{1}{2}\cdot\min\{\lfloor\mathbb{E}[X]\rfloor, c\}.$$

Proof. Let m be the median of X. By definition, $\Pr[X \geq m] \geq 1/2$. Since X is a binomial random variable, $m \geq \lfloor\mathbb{E}[X]\rfloor$ [1]. Let $m' = \min\{\lfloor\mathbb{E}[X]\rfloor, c\}$, so we have $m \geq m'$ and $\Pr[X \geq m'] \geq 1/2$.

$$\mathbb{E}[\min\{X, c\}] \geq \mathbb{E}[\min\{X, m'\}] \geq \Pr[X \geq m']\cdot m' \geq \frac{m'}{2} = \frac{1}{2}\cdot\min\{\lfloor\mathbb{E}[X]\rfloor, c\}.$$

□

Corollary E.2. *For each $k \geq 1$, let $I_1, \cdots, I_k$ be identically distributed independent binary random variables, and let $X = \sum_{i=1}^{k} I_i$. For an arbitrary constant $c \geq 1$:*

$$\mathbb{E}[\min\{X, c\}] \geq \frac{1}{4}\cdot\min\{\mathbb{E}[X], c\}.$$

Proof. If $\mathbb{E}[X] \geq 1$, then $\lfloor\mathbb{E}[X]\rfloor \geq \mathbb{E}[X]/2$ and the corollary holds by Lemma E.1. If $\mathbb{E}[X] < 1$ and $\mathbb{E}[I_i] = p$ for each $1 \leq i \leq k$, it follows that $kp = \mathbb{E}[X] < 1$.

$$\begin{aligned}
\mathbb{E}[\min\{X, c\}] \geq \mathbb{E}[\min\{X, 1\}] \geq \Pr[X = 1] &= \sum_{i=1}^{k} p(1-p)^{(k-1)} \\
&= p\sum_{i=1}^{k} e^{-1} && \text{since } kp < 1 \\
&\geq e^{-1}\cdot\mathbb{E}[X] \\
&\geq \frac{1}{4}\cdot\mathbb{E}[X] \\
&\geq \frac{1}{4}\cdot\min\{\mathbb{E}[X], c\}.
\end{aligned}$$

□

Lemma E.3. (Chernoff Bound) *Let $X_1, \cdots, X_n$ be independent trials such that for each $1 \le i \le n$, $\Pr[X_i] = p$ and $X = \sum_{i=1}^n X_i$. Then, for $\delta > 0$:*

$$\Pr[X > (1+\delta)\mathbb{E}[X]] \le \left[\frac{e^\delta}{(1+\delta)^{(1+\delta)}}\right]^{\mathbb{E}[X]}$$
$$\Pr[X < (1-\delta)\mathbb{E}[X]] \le \left[\frac{e^\delta}{(1-\delta)^{(1-\delta)}}\right]^{\mathbb{E}[X]}$$

The two bounds above imply that for $0 < \delta < 1$, we have:

$$\Pr[X > (1+\delta)\mathbb{E}[X]] \le e^{-\delta^2 \mathbb{E}[X]/3}$$
$$\Pr[X < (1-\delta)\mathbb{E}[X]] \le e^{-\delta^2 \mathbb{E}[X]/2}.$$

Lemma E.4. *Let $X_1, \cdots, X_n$ be trials such that for each $1 \le i \le n$, $\Pr[X_i \mid X_1, \cdots, X_{i-1}] \le p$. Then, the upper tail of $X = \sum_{i=1}^n X_i$ can be bounded by the upper tail Chernoff estimate for an independent set of variables $X'_1, X'_2, \ldots, X'_n$ with $\mathbb{E}[X'_i] = p$. In particular, for $0 < \delta < 1$:*

$$\Pr(X > (1+\delta)np) \le \left[\frac{e^\delta}{(1+\delta)^{(1+\delta)}}\right]^{np} \le e^{-\delta^2 np/3}.$$

Proof. Let $\vec{X}_i$ denote the shorthand for $(X_1, \ldots, X_i)$. For any $t > 0$,

$$\begin{aligned}
&\Pr(X > (1+\delta)np)\\
&= \Pr(\exp(tX) > \exp(t(1+\delta)np))\\
&\le \frac{\mathbb{E}[\exp(tX)]}{\exp(t(1+\delta)np)}\\
&= \frac{\mathbb{E}[\prod_{i=1}^n \exp(tX_i)]}{\exp(t(1+\delta)np)}\\
&= \frac{\sum_{\vec{x}_n \in \{0,1\}^n} \cdot \Pr(\vec{X}_n = x_n) \prod_{i=1}^n \exp(tx_i)}{\exp(t(1+\delta)np)}\\
&= \frac{\sum_{\vec{x}_n \in \{0,1\}^n} \Pr(\vec{X}_{n-1} = \vec{x}_{n-1}) \cdot \Pr(X_n = x_n \mid \vec{X}_{n-1}) \prod_{i=1}^n \exp(tx_i)}{\exp(t(1+\delta)np)}\\
&= \frac{\sum_{\vec{x}_{n-1} \in \{0,1\}^{n-1}} \Pr(\vec{X}_{n-1} = \vec{x}_{n-1}) \prod_{i=1}^{n-1} \exp(tx_i) \cdot \sum_{x_n \in \{0,1\}} \Pr(X_n = x_n \mid \vec{X}_{n-1}) \exp(tx_n)}{\exp(t(1+\delta)np)}\\
&\le \frac{(1+p(e^t-1)) \cdot \sum_{\vec{x}_{n-1} \in \{0,1\}^{n-1}} \Pr(\vec{X}_{n-1} = \vec{x}_{n-1}) \prod_{i=1}^{n-1} \exp(tx_i)}{\exp(t(1+\delta)np)}\\
&\le \frac{(1+p(e^t-1))^2 \cdot \sum_{\vec{x}_{n-2} \in \{0,1\}^{n-2}} \Pr(\vec{X}_{n-2} = \vec{x}_{n-2}) \prod_{i=1}^{n-2} \exp(tx_i)}{\exp(t(1+\delta)np)}\\
&\le \frac{(1+p(e^t-1))^n}{\exp(t(1+\delta)np)} \le \frac{\exp(np(e^t-1))}{\exp(t(1+\delta)np)} = \left[\frac{\exp(\delta)}{(1+\delta)^{1+\delta}}\right]^{np}
\end{aligned}$$

The last equality follows from the standard derivation of Chernoff Bound by choosing $t = \ln(1+\delta)$. ☐

References

[1] Rob Kaas and Jan M. Buhrman. Mean, median and mode in binomial distributions. *Statistica Neerlandica*, 34(1):13–18, 1980.
